# Single-Cell RNA-Seq Reveals a Population of Smooth Muscle Cells Responsible for Atherogenesis

**DOI:** 10.14336/AD.2022.0313

**Published:** 2022-12-01

**Authors:** Xiaofeng Shi, Shangming Zhu, Meijing Liu, Sara Saymuah Stone, Yao Rong, Ke Mao, Xiaopeng Xu, Chao Ma, Zhuoyuan Jiang, Yan Zha, Chun Yan, Xiaofan Yu, Di Wu, Guiyou Liu, Jidong Mi, Jianping Zhao, Yuan Li, Yuchuan Ding, Xiaogang Wang, Yong-Biao Zhang, Xunming Ji

**Affiliations:** ^1^School of Engineering Medicine, Beihang University, Beijing, China.; ^2^Beijing Institute for Brain Disorders, Capital Medical University, Beijing, China.; ^3^School of Biological Science and Medical Engineering, Beihang University, Beijing, China.; ^4^Department of Neurosurgery, Wayne State University School of Medicine, Detroit, MI, USA.; ^5^Department of Neurology and China-America Institute of Neuroscience, Xuanwu Hospital, Capital Medical University, Beijing, China.; ^6^Beijing SINOGENE Biotechnology Co., Ltd, Beijing, China.; ^7^Key Laboratory of Big Data-Based Precision Medicine (Beihang University) Ministry of Industry and Information Technology, Beijing, China.

**Keywords:** atherosclerosis, large animal model, regional susceptibility, smooth muscle cells, ScRNA-seq

## Abstract

Understanding the regional propensity differences of atherosclerosis (AS) development is hindered by the lack of animal models suitable for the study of the disease process. In this paper, we used 3S-ASCVD dogs, an ideal large animal human-like models for AS, to interrogate the heterogeneity of AS-prone and AS-resistant arteries; and at the single-cell level, identify the dominant cells involved in AS development. Here we present data from 3S-ASCVD dogs which reliably mimic human AS pathophysiology, predilection for lesion sites, and endpoint events. Our analysis combined bulk RNA-seq with single-cell RNA-seq to depict the transcriptomic profiles and cellular atlas of AS-prone and AS-resistant arteries in 3S-ASCVD dogs. Our results revealed the integral role of smooth muscle cells (SMCs) in regional propensity for AS. Notably, TNC^+^ SMCs were major contributors to AS development in 3S-ASCVD dogs, indicating enhanced extracellular matrix remodeling and transition to myofibroblasts during the AS process. Moreover, TNC^+^ SMCs were also present in human AS-prone carotid plaques, suggesting a potential origin of myofibroblasts and supporting the relevance of our findings. Our study provides a promising large animal model for pre-clinical studies of ASCVD and add novel insights surrounding the regional propensity of AS development in humans, which may lead to interventions that delay or prevent lesion progression and adverse clinical events.

Atherosclerosis (AS) is characterized by the formation of fibrofatty lesions in the tunica intima of arteries in specific regions of the vasculature [1]. AS is the leading cause of vascular diseases worldwide, followed by myocardial infarction, stroke, and gangrene [2]. The likelihood of developing AS varies markedly among different regions of the human vasculature: the abdominal, iliac, carotid, and coronary arteries are the four most AS-prone regions; whereas the subclavian, aortic arch, and thoracic arteries are AS-resistant regions [3-7]. Yet, little is known regarding the transcriptomic profiles and cellular components driving predisposition in AS-prone and AS-resistant arteries of the vascular tree. Therefore, further research is required to understand the differences in regional propensity to AS for the development of new therapeutic strategies to combat this common chronic disease.

Depicting the transcriptomic profiles and cellular atlas of AS-prone and AS-resistant arteries is challenging [1, 8-12]. It is ethically and technically difficult to isolate and obtain arteries from healthy and atherosclerotic individuals. Moreover, human atherosclerotic plaques, which are obtained from patients, who undergo endarterectomy, usually lack many smooth muscle cells (SMCs) in the tunica media and fibroblasts in the adventitia. Given the inherent shortcomings of human AS research, animal models of AS serve as an essential tool to understand the mechanisms of AS in humans [13]. However, current small animal models (mice and rabbits) and large animal models (pigs and non-human primates) of AS have many limitations, such as: (1) accelerating plaque formation by a western-type diet, (2) a different distribution of plaques as compared to humans, and (3) rare human-like endpoint events [13-15]. As such, an ideal large animal model of AS with similar pathophysiology, predilection for lesion sites, and clinical endpoint events to humans is urgently needed [15].

Dogs (*Canis familiaris*) have emerged as promising models for atherosclerosis owing to the striking similarities to humans in terms of plasma lipid profiling, cardiovascular anatomy, statin effect, and environmental effects [16, 17]. Therefore, we generated the beagle dog model for AS [18, 19], which displays hyper-cholesterolemia under normal diet (spontaneous), bears atherosclerotic burden in similar areas as human AS-prone arteries (the abdominal aorta, iliac arteries, carotid arteries, and coronary arteries), and suffers the human-like clinical complications of myocardial infarction, stroke, and disabling peripheral artery disease. Thus, this new AS model termed “3S-ASCVD dogs” serves as a powerful tool to evaluate atherosclerotic cardiovascular disease (ASCVD).

To define the transcriptomic profiles and cell atlas of AS-prone and AS-resistant arteries in the vascular tree, as well as determine the dominant cells involved in AS development, we interrogated the bulk and scRNA-seq data of AS-prone and AS-resistant arteries of 3S-ASCVD dogs. We discovered that SMCs play a dominant role in regional susceptibility to AS, and we identified the TNC^+^ SMC subset to possess remarkable phenotypic plasticity in native AS plaque formation, which may contribute to the propensity of AS development in human AS-prone arteries.

## MATERIALS AND METHODS

### Ethical guidelines

This study was approved by the Ethics Committee of the School of Biological Science and Medical Engineering, Beihang University, Xuanwu Hospital Capital Medical University, and Beijing SINOGENE Biotechnology Co., Ltd, in compliance with the Declaration of Helsinki Principles. Eligible subjects gave informed written consents.

### Large animal model for AS

The 3S-ASCVD dog, named “Apple”, is an ApoE gene knockout dog generated by the CRISPR/Cas9 system [18]. The ear skin fibroblasts of “Apple” were used as donor cells for somatic cell nuclear transfer to produce cloned beagle dogs named “Xixi” and “Nuonuo”, respectively [19]. All three dogs were fed a normal diet and raised at Beijing SINOGENE Biotechnology Co., Ltd. Serum biochemical analyses were conducted, and AS plaques at multiple sites were assessed via ultrasound-based imaging methods. To avoid data variation incurred by sex differences, three age-matched male wild type (WT) beagle dogs served as controls. All animal experiments were supervised by two certified veterinarians.

### Human carotid atherosclerotic tissue specimens

Three symptomatic male patients (64, 69 and 62 years) undergoing carotid endarterectomy at Xuanwu Hospital Capital Medical University were enrolled in our study. The three patients were negative for infectious disease, autoimmune disease, or cancer. Carotid atherosclerotic tissue specimens were obtained from the carotid bifurcation point and immediately placed in a tissue storage solution (Miltenyi Biotec, Bergisch Gladbach, Germany) on ice.

### Liver sample preparation, iTRAQ labeling, and peptide separation

The liver samples were harvested from WT (n=3) and 3S-ASCVD dogs (n=3). The liver samples were subsequently homogenized with lysis buffer (7 M urea, 2 M thiourea, 0.1% CHAPS, Protease Inhibitor Cocktail) and total protein was extracted. Insoluble debris was pelleted by centrifugation at 13000 rpm for 15 min at 4°C. The supernatant protein was quantified by the Bradford Assay kit (BioRad, Hercules, CA) using bovine serum albumin as the standard. Subsequently, the protein sample was digested overnight at 37? with trypsin (a protein to enzyme ratio of 50:1, w/w) and labeled with isobaric tags for relative and absolute quantitation reagents (iTRAQ) (Applied Biosystems, Foster City, CA) according to the manufacturer's protocol. The labeled peptides were then dried by speed vacuuming and stored at -20°C.

### Mass spectrometric analysis

The lyophilized fractions were redissolved in 0.1% formic acid. Each sample was injected onto Easy 1000 nanoLC (Thermo-Fisher Scientific, San Jose, CA) for preconcentration and cleanup. Reverse phase separation of the labeled peptide mixture was performed in a low-pH nanoLC system using a C-18 column (150 µm i.d. 250 mm length, 1.9 µm particles). The solvent system was as follows: solvent A was 0.1% (w/v) formic acid/H_2_O, and solvent B was 0.1% (w/v) formic acid/H_2_O and 99.9% (v/v) acetonitrile. The sample was eluted with a linear gradient from 6% solvent B to 35% solvent B over 90 minutes with a flow rate of 0.6 µL/min. All mass spectrometric analyses were performed on an Orbitrap Fusion Lumos mass spectrometer (Thermo-Fisher Scientific, San Jose, CA). Mass spectrometric spectra were acquired in the 350-1550 m/z range using a mass resolution setting of 120,000.

### Database searching and iTRAQ quantification

All mass spectrometric spectra were generated and analyzed using Mascot Daemon (Matrix Science, London, U.K.; version 2.5.1) by default setting. In the present study, we used the UniProt *Canis lupus familiaris* database with 45350 entries, and we assumed that the digestion enzyme was trypsin with a maximum of two missed cleavages allowed. Methionine oxidation and Acetyl (N-terminal) were specified as variable modifications for protein identification. Moreover, the mass tolerance parameters for peptide identification were ±10 ppm for precursor ions and ±0.05 Da for fragment ions.

### Systemic arterial dissection and collection

To ensure successful isolation, samples were collected across 6 days allowing that only one specimen was processed per day. Animals were dissected, including three 3S-ASCVD dogs (~2 years old) and three WT beagle dogs (~2 years old). Each dog underwent surgically isolated arterial segmentation from seven regions: coronary arteries, carotid arteries, iliac arteries, subclavian arteries, and the aorta (including aortic arch, thoracic aorta, and abdominal aorta). Dissected arterial segments were stored in liquid nitrogen for further bulk gene expression analysis. In addition, surgically excised iliac arteries and subclavian arteries were stored on ice in 1ml RPMI media (Gibco, Grand Island, NY, USA) containing 10% fetal bovine serum (Gibco, Grand Island, NY, USA) for subsequent enzyme digestion and single cell RNA sequencing (scRNA-seq).

### Bulk RNA-seq and differential gene expression analysis

Dissected arterial segments were lysed in Trizol reagent (Invitrogen, CA, USA) for RNA isolation. To perform bulk RNA-seq, the quantity and quality of extracted RNA was assessed on the NanoDrop Spectrophotometer (NanoDrop Technologies, Wilmington, DE, USA) and the Agilent 2100 Bioanalyzer (Agilent, Santa Clara, CA, USA). Sequencing libraries were constructed using the TruSeq Stranded mRNA Library Prep Kit (Illumina, USA) and sequenced on the Novaseq 6000 platform (Illumina, USA) with 150 bp pair-ends reads. After removing contaminated and low-quality reads, high-quality clean reads were aligned to the *Canis lupus familiaris* genome (canFam3) using HISAT2 [20]. Subsequently, uniquely aligned reads were assembled and quantified using StringTie [21]. The differentially expressed genes(DEGs)were identified using the R package DESeq2 [22]. The adjusted P<0.01 and |log2(fold change)|>1 were used as cut-off criteria for DEGs.

### Tissue digestion and cell dissociation for scRNA-seq

Both the iliac and subclavian arteries were washed three times with pre-cooled RPMI 1640 media (Gibco, Grand island, NY, USA), cut into small pieces (~1 mm^3^), and digested for 1 hour at 37? in Hanks’ Balanced Salt solution (Sigma, St. Louis, MO, USA) containing 450U/ml Collagenase I (Sigma, St. Louis, MO, USA), 250U/ml Collagenase XI (Sigma, St. Louis, MO, USA), 120U/ml Hyaluronidase (Sigma, St. Louis, MO, USA) and 120U/ml DNase I (Sigma, St. Louis, MO, USA). The digested cell suspension was successively filtered through a 70 µm and 40 µm cell strainer (Miltenyi Biotec, Bergisch Gladbach, Germany), followed by washing with 37°C pre-warmed RPMI media (Gibco, Grand Island, NY, USA) (500 g, 5 min). Red blood cell contaminants were lysed with RBC lysis solution (Miltenyi Biotec, Bergisch Gladbach, Germany) and cell debris removed (Miltenyi Biotec, Bergisch Gladbach, Germany) according to the manufacturer's instructions.

Human carotid atherosclerotic plaques were processed within 1 hour of surgery. Each specimen was extensively washed in RPMI 1640 media, weighed, and digested at 37 °C for 1h in RPMI media containing 1mg/ml Collagenase I (Sigma, St. Louis, MO, USA), 1mg/ml Collagenase III (Solarbio, Beijing, China), 1mg/ml Collagenase IV (Sigma, St. Louis, MO, USA), 1mg/ml Collagenase V (Sigma, St. Louis, MO, USA) and 10 mM CaCl_2_. The mixture was successively filtered through 70 μm, 40 μm and 30 μm cell strainers, washed twice in PBS, and centrifuged at 200g for 5 min. Dead cells were removed with Dead Cell Removal Kit (Miltenyi Biotec, Bergisch Gladbach; Germany) according to the manufacturer’s instructions. Cells were counted with an automatic cell counter (Counstar, Beijing, China).

### ScRNA-seq and raw data preprocessing

Single cells were encapsulated in droplets using 10× Genomics GemCode Technology following the manufacturer’s specifications. ScRNA-seq libraries were generated using the Single Cell 3’ Solution v3 Reagent Kit (10× Genomics). Sequencing was performed on the Illumina NovaSeq 6000 sequencer with a sequencing depth of at least 100,000 reads per cell and 150 bp (PE150) paired-end reads. 10× genomics single cell transcriptome sequencing data was processed using CellRanger Single Cell software suite Version 3.1.0 (https://support.10xgenomics.com) as previously described [23]. The reference genome for the dogs was canFam3 and the reference genome for the humans was GRCh38.

### Cell clustering and cell marker identification

Cell clustering was performed using R package Seurat v3 [24]. All functions were run with default parameters unless otherwise specified. To ensure quality of input data, genes expressed in fewer than 3 cells were removed. Cells with gene number > 200 and < 4000 and mitochondrial genes ratio < 0.2 were maintained for downstream analysis. The remaining data was normalized and log-transformed, and the highly variable genes were detected. Principle component analysis (PCA) was used for dimensionality reduction. After calculation with the built in “JackStraw” function, the first 20 significant principal components were considered for clustering analysis. Cluster detection was run with resolution of 0.5. T-distributed stochastic nearest neighbor embedding (t-SNE) was used for data visualization in two dimensions. The DEGs were identified using the “FindMarkers” or “FindAllMarkers” function in the Seurat package. Cell markers of different clusters were selected from DEGs with adjusted *P* < 0.05 and log (fold change) > 0.5. In the present study, we only extract the non-immune cells (PTPRC-) subset for subsequent analysis.

### RNA velocity analysis and probabilistic fate mapping

We performed RNA velocity analysis as described by La Manno *et al* [25]. Scvelo was employed to obtain the spliced and unspliced transcript reads from individual cells and infer transitional statistics. After normalization, log-transformation and first- and second- order moments calculations for each cell occurred across its nearest neighbors, and the velocities for each cell was computed. Next, the velocities of cells were visualized to the top of the previously calculated t-SNE plot generated by Seurat. Moreover, we used the CellRank function to uncover cellular dynamics and compute its fate probability of it reaching any of the terminal states based on scRNA-seq data with RNA velocity annotation.

### Constructing cell trajectories of cells along the pseudotime

The R package Monocle2 (version 2.9) was applied to construct single-cell trajectories of specific cell types from SMCs to MFB [26]. The first 1000 (by *P*) DEGs in each cluster were used for pseudotime ordering by function differential GeneTest. The DDRTree algorithm was utilized for dimension reduction and cell ordering along the pseudotime trajectory.

### Ligand-receptor cellular communication analysis

All gene expression data of labeled cells was used as the input data, and the R package CellChat (https://github.com/sqjin/CellChat) was used to infer, visualize, and analyze the intercellular communications [27]. The ligand-receptor interaction database contained 2,021 validated molecular interactions, which were further classified into 229 functionally related signaling pathways. CellChat identified differentially over-expressed ligands and receptors for each cell group. Finally, the significant cellular communications were identified and visualized by circle plot.

### Biological functional interpretation of DEGs

The online based software programs DAVID (https://david.ncifcrf.gov/) and GeneCodis 4.0 (https://genecodis.genyo.es/) [28] were used to elucidate the biological functional interpretation of DEGs. The listed target genes can be mapped to associated Gene Ontology (GO) terms.

Similarly, the target genes were also assigned Kyoto Encyclopedia of Genes and Genomes (KEGG) terms and an enrichment analysis was performed. Because most lists of GO terms are large and redundant, REVIGO was applied to summarize redundant GO terms based on semantic similarity measures [29]. Nonredundant GO term sets were visualized.

**Figure 1.**
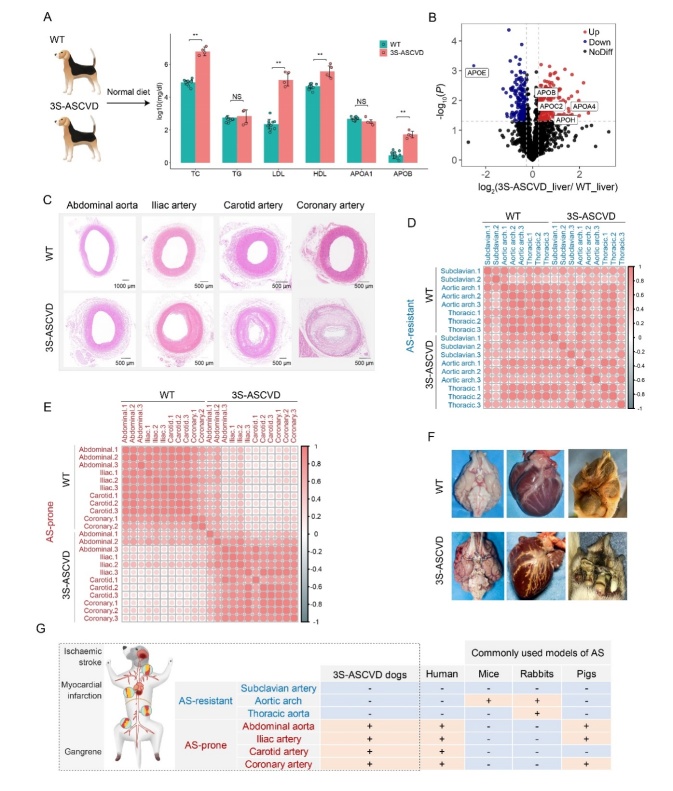
3S-ASCVD dogs represent a promising large animal model for AS. (A) Change of serum lipid levels in 3S-ASCVD dogs (n=4) compared with WT (wild type) dogs (n=10) fed on a normal diet (NS: no significant, **P<0.01 vs. WT dogs by wilcoxon test). TC, total cholesterol; TG, triglyceride; LDL-C, low density lipoprotein cholesterol; HDL-C, high density lipoprotein cholesterol; APOA-1, apolipoprotein A1; APOB, apolipoprotein B. (B) Volcano plot showing up-regulated (red color) and down-regulated proteins (blue color) in the liver of 3S-ASCVD dogs (n=3). The apolipoproteins showing significant differences in expression (*P*<0.05, |fold change|>1.2) are indicated. APOE protein expression was significantly down-regulated in livers of 3S-ASCVD dogs, confirming the knockout of APOE gene. (C) Representative image of histopathology showing area of stenosis in abdominal aorta, iliac arteries, carotid arteries and coronary arteries of 3S-ASCVD dogs compared to WT dogs. (D) Pearson correlation analysis illustrating the similarity of gene expression profiles of AS-resistant arteries obtained from WT and 3S-ASCVD dogs. (E) Pearson correlation analysis illustrating the heterogeneity of gene expression profiles of AS-prone arteries obtained from WT and 3S-ASCVD dogs. (F) Figure illustrating the myocardial damage, cerebral changes and gangrene in 3S-ASCVD dogs. (G) The major advantages of 3S-ASCVD dogs. 3S-ASCVD dogs developed AS with similarities to humans in terms of specific predisposition for lesion sites and clinical endpoint events.

### RNA extraction and qRT-PCR

The AS-prone and AS-resistant arteries in WT dogs (n=3) were prepared for total RNA extraction. Total RNA was extracted with TRIZOL reagent according to manufacturer’s recommendations. One microgram of total RNA from each sample was used to synthesize first-strand cDNA using M-MLV reverse transcriptase (Promega) as the protocol described. qRT-PCR was performed with 10 ng RNA and the reverse transcription kit Toyobo SYBR Green following manufacturer’s guidelines. All samples were analyzed in triplicate. The 2^-ΔCt^ method was used to calculate the relative fold gene expression and GAPDH was used as reference gene.

### RNA fluorescence in-situ hybridisation (FISH)

The AS-prone iliac arteries were used for RNA-FISH. After dehydration of the artery tissues by gradient alcohol, paraffin and embedding, the paraffins were sliced, dewaxed and dehydrated. The slices were boiled in the retrieval solution for 10-15 minutes and naturally cooled. The objective tissue was marked and added with proteinase K (20 μg/ml) working solution to cover objectives at 37°C. Pre-hybridization solution was added to each section and incubated for 1 h at 37°C. Then the probe hybridization solution was added in the section and incubation in a humidity chamber and hybridize overnight. The sections were washed in 2×SSC (Servicebio, G30164) for 10 min at 37°C, 1×SSC two times for 5 min each at 37°C, and wash in 0.5×SSC for 10 min at room temperature. Finally, cell nuclei were stained with DAPI for 8 min in the dark. Microscopic examination and photography were performed with positive fluorescence microscope.

### Statistical Analysis

The analysis is carried out in the R environment for statistical computing and visualization. The non-parametric wilcoxon test was used for comparison of two groups with less than 30 samples in each group. Data were expressed as mean±SD. Statistical significance was accepted for *P*<0.05. For differential proportion, differential expression, and enrichment analyses, *P* were adjusted for multiple hypothesis testing using the Benjamini-Hochberg method. The number of samples used for each analysis is indicated in the figure legends.

## RESULTS

3S-ASCVD dogs represent an ideal spontaneous large animal model for AS

To develop a large animal model that reliably and precisely mimics human AS development, we created the 3S-ASCVD dogs. The 3S-ASCVD dogs were ApoE gene knockout dogs generated by the CRISPR/Cas9 system. After 18 months on a normal diet, serum lipids were measured revealing that total cholesterol (TC), low density lipoprotein (LDL), high density lipoprotein (HDL), and apolipoprotein B (APOB) levels were substantially elevated in 3S-ASCVD dogs compared to WT dogs (Fig. 1A). The abnormal lipid levels indicated that 3S-ASCVD dogs developed marked hypercholesterolemia. The results of the liver proteome further confirmed the abnormalities in lipid metabolism (Supplementary Table 1 and Fig. 1B). The abnormalities of hepatic lipid metabolism in 3S-ASCVD dogs was further elucidated through detection of significantly increased expression levels of apolipoproteins involved with LDL transport including APOA4, APOB, APOC2, and APOH (Fig. 1B).

Next, we evaluated whether the 3S-ASCVD dogs developed AS similar to humans and reflected specific predisposition for lesion sites and clinical endpoint events. We performed ultrasound and angiography on 3S-ASCVD dogs and observed plaque formation in the four human AS-prone arteries (abdominal aorta, iliac arteries, carotid arteries, and coronary arteries) (Supplementary Fig. 1A and 1B). Furthermore, we did not detect plaques in the three AS-resistant arteries (subclavian arteries, aortic arch, and thoracic aorta) (data not shown). Histopathology further confirmed the presence of plaques in the four AS-prone arteries (Fig. 1C). Notably, area stenosis was highest in coronary arteries and carotid arteries, followed by iliac arteries and abdominal aorta (Fig. 1C). By surveying the gene expression changes of four AS-prone arteries (Supplementary Table 2 and Supplementary Fig. 2A-2F), we confirmed that AS is a lipid-driven chronic inflammatory disease [2] and an ongoing stiffness disease [30]. Our analysis revealed a significantly positive correlation between the AS-resistant arteries from 3S-ASCVD dogs and the corresponding arteries from WT dogs (Fig. 1D), but no significant correlation between both groups for the AS-prone arteries (Fig. 1E). Moreover, the 3S-ASCVD dogs suffered human-like spontaneous (without other interventions) endpoints, such as myocardial infarction, transient ischemic attack or lower limb gangrene due to AS obliterans (Fig. 1F and Supplementary Movie 1). All these features were present in 3S-ASCVD dogs, making it a promising large animal model of AS which can be widely used in pre-clinical medical and pharmaceutical studies of atherosclerotic cardio-cerebral vascular diseases (Fig. 1G).

**Figure 2.**
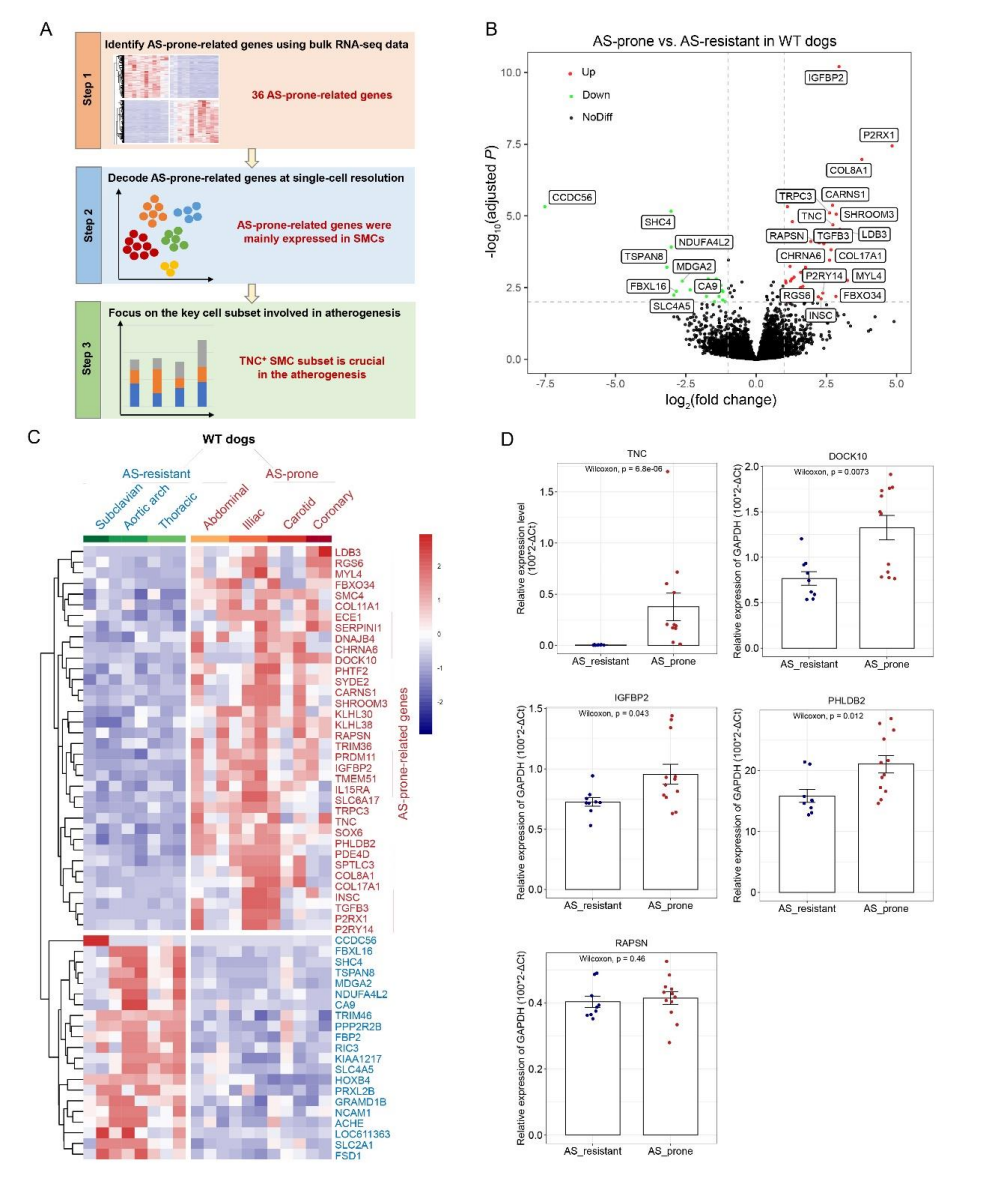
Transcriptomic profiles of AS-prone arteries and AS-resistant arteries. (A) Workflow of identification of AS-associated cell populations. (B) Volcano plot showing the DEGs in AS-prone arteries compared with AS-resistant arteries in WT dogs. Genes showing significant differences in expression (adjusted *P*<0.01, log_2_(fold change)>2) are indicated. (C) Heatmap showing the specific gene expression signature of AS-prone arteries. (D) The expression of key AS-prone-related genes were validated in AS-resistant arteries (n=9) and AS-prone arteries (n=12) from three WT dogs by qRT-PCR (*P* was calculated by non-parametric wilcoxon test).

### Specific gene expression signature of AS-prone arteries in WT dogs

To better identify the AS-associated cell populations, we applied bulk RNA-seq in combination with single-cell RNA-seq analysis of AS-prone and AS-resistant arteries to elucidate both the subtleties and complexities of regional differences in AS susceptibility (Fig. 2A). Since the artery regions susceptible to AS are non-random, there may be clues for increased expression of AS-prone-related genes in the high-susceptibility regions before lesion development. We compared the inherent regional signature of AS-prone arteries and AS-resistant arteries in WT dogs. Overall, we identified 36 up-regulated DEGs (AS-prone-related genes) and 21 down-regulated DEGs (Supplementary Table 3), with the most significant DEGs shown in Fig. 2B. The specific gene expression signature of AS-prone arteries is shown in Fig. 2C. Of note, we did not find increased expression of immune-related genes, which implied that dysregulation of non-immune genes might determine the non-random regional susceptibility to AS.

qRT-PCR was carried out to verify the aforementioned AS-prone-related genes. The results revealed that the expressions of TNC, DOCK10, IGFBP2, PHLDB2 and RAPSN were higher in AS-prone arteries compared with AS-resistant arteries in WT dogs, which was consistent with the bulk RNA-seq data (Fig. 2D). Importantly, the higher expression of TNC was most significant. Moreover, the genetic association between polymorphisms in TNC and AS disease has been previously reported [31, 32]. TNC, a highly conserved, multifunctional extracellular matrix glycoprotein of tenascin-C [33], is tightly controlled in adults and displays co-incidence with sites of vascular disease in adult arteries [34]. TNC can promote the proliferation and migration of SMCs, which play a vital role in atherosclerotic plaque development [35]. Moreover, the expression level of TNC correlates with the degree of inflammation present in human atherosclerotic plaques [34]. Our whole tissue “bulk” sequencing emphasized the importance of non-immune-related genes in regional susceptibility to AS, such as TNC. We next unraveled the heterogeneity in healthy and atherosclerotic vasculature at the single-cell level and focused on the potential role of non-immune cells in the regional propensity differences of AS development.

### Cellular composition changes in AS-prone and AS-resistant arteries

**Figure 3.**
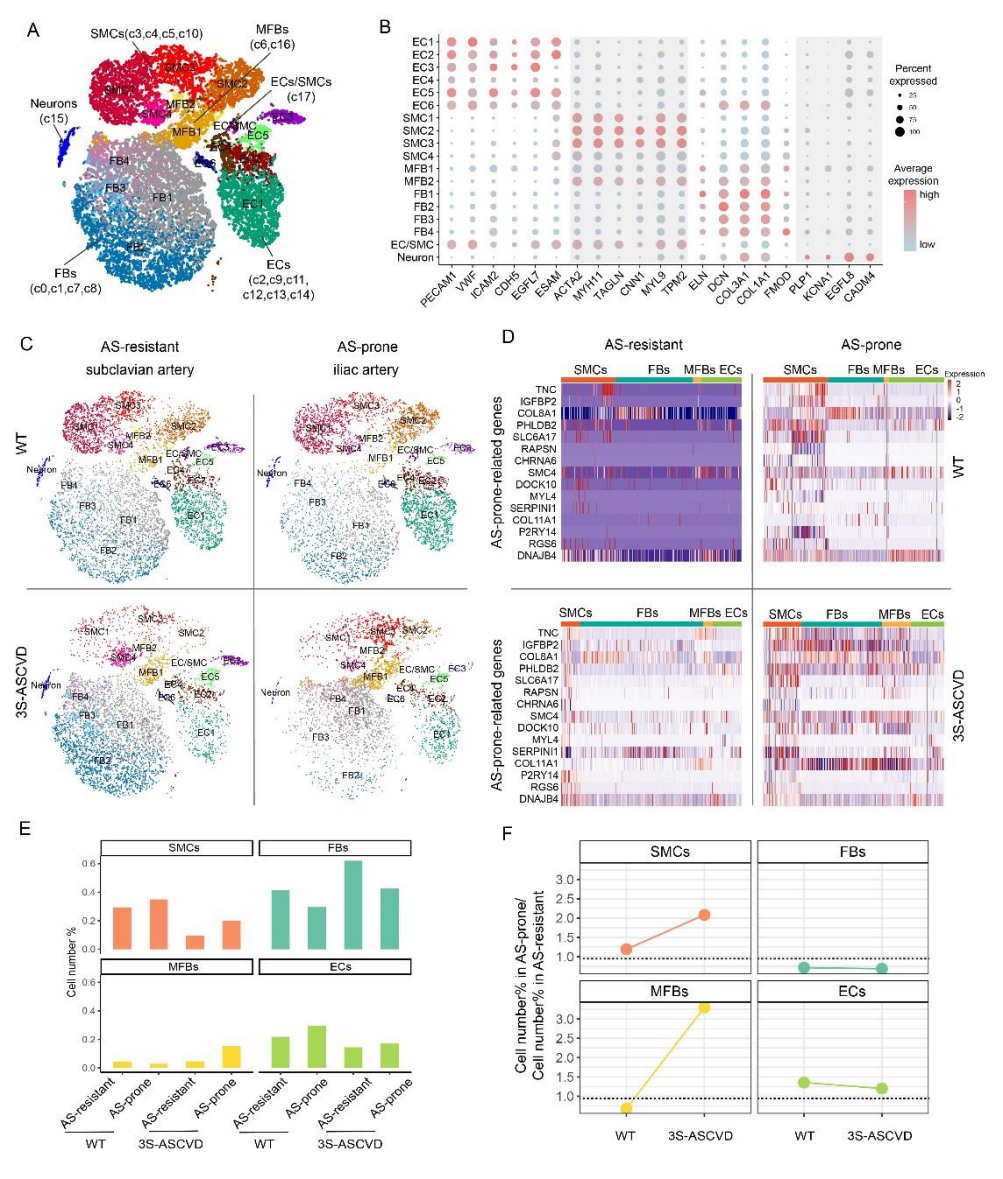
Single cell landscape of AS-prone arteries and AS-resistant arteries. (A) The major cell clusters in a t-SNE diagram. ECs, endothelial cells; SMCs, smooth muscle cells; FBs, fibroblasts; MFBs, myofibroblasts. (B) The canonical cell markers defining each type of cell cluster are listed. Gray bars are added to aid interpretation. (C) The major cell clusters in AS-prone arteries and AS-resistant arteries from WT and 3S-ASCVD dogs, respectively. (D) Heatmap of the AS-prone-related genes in AS-prone and AS-resistant arteries. (E) Bar chart of the relative proportion of the major cell types in AS-prone arteries and AS-resistant arteries from WT and 3S-ASCVD dogs. (F) The change in the relative proportion of the major cell types in AS-prone arteries vs. AS-resistant arteries in WT and 3S-ASCVD dogs.

To better understand the regional differences in AS susceptibility, we performed scRNA-seq analyses on the non-immune cells of AS-prone and AS-resistant arteries from WT and 3S-ASCVD dogs. To efficiently detect the difference of cellular composition and the transcriptome changes in early-stage lesions, we isolated AS-prone iliac arteries according to the degree of vascular stenosis. We also simultaneously isolated AS-resistant subclavian arteries with comparable artery diameter to the iliac artery. Due to the sudden death of two 3S-ASCVD dogs in the F1 generation and poor cell activity, we only obtained scRNA-seq data of one AS-prone iliac artery and AS-resistant subclavian artery from one 3S-ASCVD dog. In total we obtained 29,517 high-quality cells and detected an average number of 1,402 genes per cell (Supplementary Table 4). The non-immune cells were grouped into 18 clusters corresponding to smooth muscle cells (SMCs), fibroblasts (FBs), myofibroblasts (MFBs), endothelial cells (ECs), and neurons by canonical cell markers (Fig. 3A and 3B). The cell composition was consistent with the anatomical regions of the tunica intima, media, and adventitia of arterial vessels [36]. Our results further supported that SMCs and FBs were dominant in arterial vessels [37]. Moreover, we observed a very similar cellular landscape of AS-prone and AS-resistant arteries in WT dogs, but a very distinct cellular landscape of AS-prone and AS-resistant arteries in 3S-ASCVD dogs, consistent with the transcriptome in bulk vascular tissues (Fig. 3C).

To determine the dominant cell populations which control the specific gene expression signature of AS-prone arteries, we decoded the AS-prone-related genes at the single-cell resolution. Briefly, 15 of the 41 up-regulated AS-prone-related genes were detected in the single cell data, and heatmaps indicated that SMCs seem to dominate the specific gene expression signature of AS-prone arteries (Fig. 3D). By comparing the percentage of the major cell types in AS-prone and AS-resistant arteries, we found that the AS-prone artery had a higher proportion of SMCs in both WT and 3S-ASCVD dogs (Fig. 3E and 3F), which suggested that SMCs may be closely related to the regional susceptibility to AS and play an important role in onset of atherosclerosis [38]. Meanwhile, we observed a higher proportion of MFBs in the AS-prone artery than AS-resistant artery in 3S-ASCVD dogs, while such phenomenon was not observed in WT dogs (Fig. 3E and 3F). Altogether, our results suggested that SMCs play a much more complex role in regional susceptibility to AS and is accompanied by a significant increase of MFBs in atherogenesis.

### TNC^+^ SMC subset is crucial in the atherogenesis of AS-prone arteries

**Figure 4.**
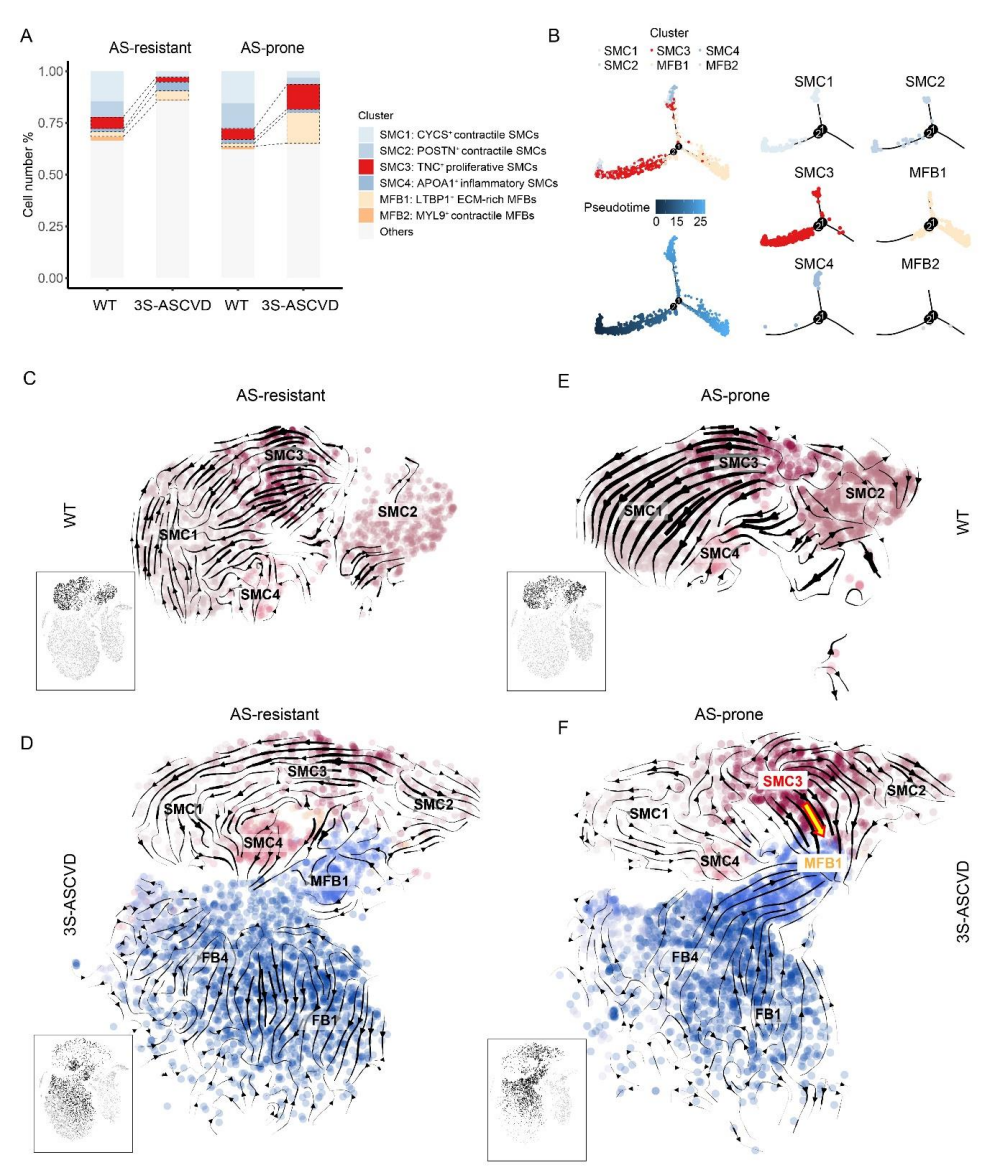
TNC^+^ SMC subset is crucial in the atherogenesis of AS-prone arteries. (A) Bar chart of the relative proportion of SMC subsets and MFB subsets in AS-prone arteries and AS-resistant arteries from WT and 3S-ASCVD dogs. (B) Pseudotime analysis showing transdifferentiation of SMC to MFB. (C-F) RNA velocity analysis illustrating the extent and direction of cell differentiation of SMC subsets in the t-SNE plot. The direction of the arrow represents the future state of the cells. Velocity field superimposed to the t-SNE embedding of cells by sample. Cells are colored by cell type according to t-SNE uniform manifold approximation and projection.

To identify the key cell subset involved in atherogenesis of AS-prone arteries, we further analyzed the changes in cell subset proportions of SMCs and MFBs. In total, the 3S-ASCVD dogs had a decrease in the proportion of SMCs and had an increase in the proportion of MFBs (Fig. 4A). Surprisingly, we found that the proportion of TNC^+^ SMC subset was uniquely increased in the AS-prone artery (Fig. 4A, Supplementary Table 5 and Supplementary Fig. 3), suggesting that the TNC^+^ SMC subset played a sophisticated role in modulating atherogenesis of AS-prone arteries. Meanwhile, we observed an increase in the proportion of the LTBP1^+^ MFB subset in the AS-prone artery (Fig. 4A, Supplementary Table 6 and Supplementary Fig. 4). Trajectory analysis suggested the transdifferentiation of SMCs to MFBs (Fig. 4B). It is well accepted that SMCs from the media serve as an important origin of MFBs, which acquires the phenotypic features of MFBs during the migratory and replicative process occurring in plaque [39, 40]. Thus, we postulated that TNC^+^ SMC subset is crucial in the atherogenesis of AS-prone arteries, which would be a major origin of the LTBP1^+^ MFB subset.

To test the hypothesis that TNC^+^ SMC subset is the major origin of LTBP1^+^ MFB subset in the atherogenesis of AS-prone arteries, we performed RNA velocity analysis. In general, we observed similar transcriptional dynamics of AS-prone and AS-resistant arteries in WT dogs (Supplementary Fig. 5A and 5B), but a very distinct transcriptional dynamic of AS-prone and AS-resistant arteries in 3S-ASCVD dogs (Supplementary Fig. 5C and 5D). Significantly, the stream plots showed that the AS-prone artery has a main direction of flow from cells in the TNC^+^ SMC subset to MFB subset in 3S-ASCVD dogs (Fig. 4C-4F). The RNA FISH results showed that ACTA2, TNC and COL3A1 are expressed in the fibrous cap of atherosclerotic plaque, suggesting the role of TNC+ SMCs in atherogenesis (Supplementary Fig. 6A and 6B). This flow employs LTBP1, a latent TGFβ binding protein [41], as a key driver gene (Supplementary Fig. 7A). Once again, these data suggested that TNC^+^ SMC subset is the originator of LTBP1^+^ MFB subset. SMC-derived cells possess remarkable ability to dynamically modulate their phenotype, and are proposed to generate 30-70% of all plaque cells and play central roles in plaque development, progression and stability [42, 43]. Together with our finding that TNC^+^ SMC subset can differentiate into the LTBP1^+^ MFB subset in AS-prone arteries, we proposed that the TNC^+^ SMC subset is most likely to be the “pioneer” cells recruited from the media to undergo phenotypic conversion and hence promote the development of AS.

**Figure 5.**
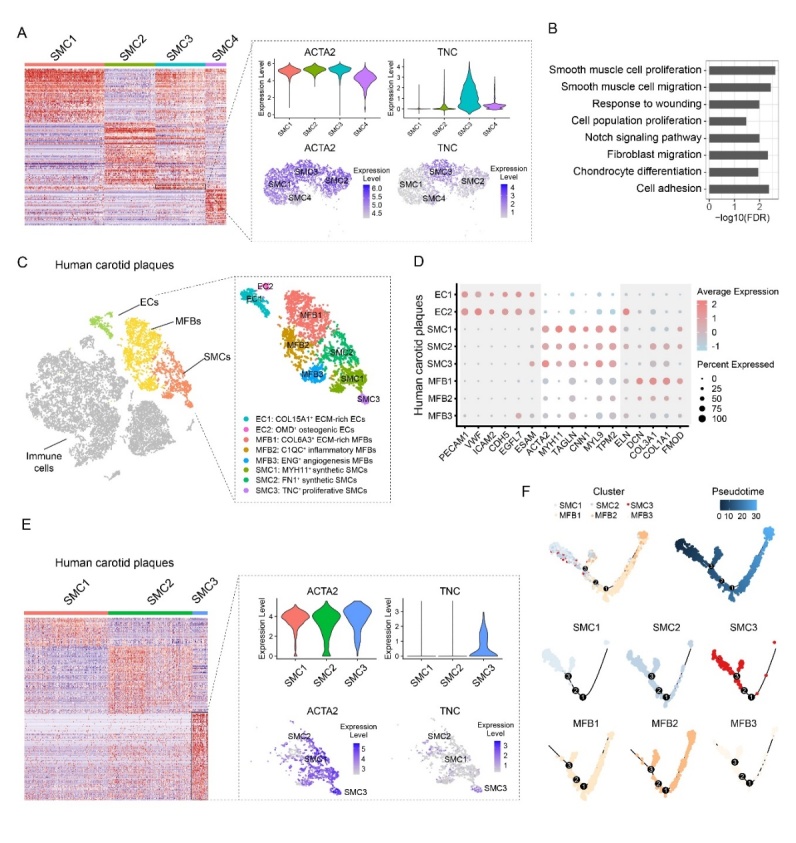
Activated TNC^+^ SMC subset is involved in formation of human AS-prone carotid plaques. (A) Heatmap of hierarchically clustered highly variable genes across SMCs in beagle dogs. Rows show Z-scored gene expression values, and columns show individual cells. The dotted box (right) listed the feature genes found in cells of TNC^+^ SMC subset. (B) The enriched GO terms of highly variable genes in cells of TNC^+^ SMC subset in beagle dogs. (C) The major cell clusters of human carotid plaques (n=3) in a t-SNE diagram. ECs, endothelial cells; SMCs, smooth muscle cells; MFBs, myofibroblast. The cell clusters of non-immune cells are shown in the right dotted box. (D) The dot plot of canonical cell markers for non-immune cells of human carotid plaques. (E) Heatmap of hierarchically clustered highly variable genes across SMCs in human carotid plaques. Rows show Z-scored gene expression values, and columns show individual cells. The dotted box (right) lists the featured genes found.

### TNC^+^ SMC subset exhibits an activated phenotype

To define the function of TNC^+^ SMC subset, we performed subset-specific transcriptome analysis and found that TNC^+^ SMC subset displayed transcriptional signatures associated with response to wounding, smooth muscle cell proliferation, smooth muscle cell migration, fibroblast migration, and chondrocyte differentiation (Fig. 5A and 5B). By cell-cell communication analysis, we further identified TNC^+^ SMC subset as a dominant communication “hub” (Supplementary Fig. 7B). Our results suggested that the TNC^+^ SMC subset exhibited an activated phenotype which was characterized by a high degree of self-proliferation, plasticity, and cell communication.

To understand the mechanisms by which the TNC^+^ SMC subset contributes to atherogenesis in AS-prone arteries, we next deciphered TNC^+^ SMC subset-specific transcriptional changes. We identified 11 up-regulated DEGs (Supplementary Table 7), and the most significant up-regulated DEGs (POSTN, VCAN, and FBN1) are shown in Supplementary Fig. 7C and Supplementary Fig. 7D. POSTN, an epithelial-to-mesenchymal transition hallmark gene [44], plays an important role in stem cell maintenance [45]. VCAN is a major component of the extracellular matrix involved in cell adhesion, proliferation, and migration [46]. FBN1 acts as an extracellular matrix structural constituent [47]. Taken together, our results suggest that TNC^+^ SMC subset increases extracellular matrix deposition and remodeling, which will promote atherogenesis in AS-prone arteries.

### TNC^+^ SMC subset is involved in formation of human AS-prone carotid plaques

Given that SMCs infiltrate within lesions during atheromatous plaque formation to undergo proliferation and phenotypic changes [48]; we reasoned that the activated TNC^+^ SMC subset is involved in formation of human AS-prone carotid plaques. Therefore, we performed scRNA-seq analysis on the human AS-prone carotid plaques (n=3 patients). The major non-immune cell types in human carotid plaques were grouped into 8 clusters corresponding to SMCs, MFBs and ECs by the same canonical cell markers used for beagle dogs (Fig. 5C and 5D). The carotid plaques lacked FBs and have a small proportion of SMCs. This is explained by loss of FBs in the adventitia and SMCs in the media layer in human carotid plaques obtained from endarterectomy (Fig. 5C and 5D). Of note, TNC was specifically expressed in one of the three SMC subsets (TNC^+^ SMC) in human carotid plaques (Fig. 5E and Supplementary Table 8). The pseudotime result suggested that the TNC^+^ SMC subset can transdifferentiate into MFBs (Fig. 5F). Overall, our data suggested that TNC^+^ SMC subset can infiltrate within plaque lesions, which is involved in formation of human AS-prone carotid plaques.

## DISCUSSION

The use of ideal large animal models of AS is crucial for clarifying the disease mechanism and advancing pharmaceutical research [14]. In the current study, we reported that 3S-ASCVD dogs represent a valuable spontaneous large animal model for AS. Here, we expose the advantages of the 3S-ASCVD dogs: (1) having close similarities to humans in terms of genetic, metabolic, physiological, and anatomical characteristics [17], (2) developing atherosclerotic lesions in a spontaneous manner after consumption of a normal diet, (3) sharing the topography of the atherosclerotic lesions with humans including abdominal, iliac, carotid, and coronary arteries, and (4) suffering from human-like clinical complications of cardiovascular, cerebrovascular, and peripheral artery diseases. Hence, 3S-ASCVD dogs should be validated models for pre-clinical medical and pharmaceutical studies of atherosclerotic cardio-cerebral vascular diseases.

SMCs comprise the primary cell type in all stages of atherosclerotic plaque progression, which are located in the medial layer of arteries and retain remarkable plasticity (shifting from a differentiated contractile phenotype to a dedifferentiated synthetic phenotype) [30, 36, 49]. Accumulating evidence has shown that the major determinant for regional propensity or resistance to AS is the developmental origins of SMCs such as neural crest, proepicardium, mesothelium, secondary heart field, somites, mesoangioblasts and different types of self-renewing progenitor cells [36, 38, 50-52]. However, the four most AS-prone arteries (abdominal, iliac, carotid, and coronary arteries) are heterogeneous in the embryonic origins for SMCs [38], which suggest there must be consistent patterns of adaptive or pathologic responses characteristic in the four most AS-prone arteries of the mature vascular tree. The inherent regional signature of the four AS-prone arteries in adult WT beagle dogs revealed the indispensable role of non-immune genes (ie. TNC) in regional propensity to AS. It has been reported that the polymorphic variants in TNC are associated with AS [31], and the expression of TNC is tightly controlled in adults and co-incident with sites of vascular disease in adult arteries [34]. High expression of TNC in the four AS-prone arteries is most likely to be the molecular basis of regional propensity to AS. Our scRNA-seq results further indicated that SMCs play a dominant role in controlling the specific gene expression signature of AS-prone arteries, which re-interpreted the meaning of SMCs in regional propensity to AS at the single-cell level. Moreover, the TNC^+^ SMC subset was markedly increased in AS-prone arteries and exhibited an activated phenotype (stronger ability of proliferation, migration, and differentiation); which suggest the activation of TNC^+^ SMC subset is a hallmark of AS and contributed to the onset of atherosclerotic lesion in AS-prone arteries of 3S-ASCVD dogs. The single cell data for human carotid plaques also supported the role of TNC^+^ SMC subset in the pathological process of plaques.

Taken together, we reported that 3S-ASCVD dogs represent an ideal animal model for AS. Our findings add novel insights to the complexity of SMCs in the regional propensity to AS and reveal a novel cellular target (TNC^+^ SMC subset) for early intervention of AS.

## Supplementary Materials

The Supplementary data can be found online at: www.aginganddisease.org/EN/10.14336/AD.2022.0313.
