# Supplementary material for: Single-Cell RNA-Seq Reveals a Population of Smooth Muscle Cells Responsible for Atherogenesis
Source: Aging Dis. 2022 Dec 1;13(6):1939–53. doi: 10.14336/AD.2022.0313 (PMC9662277; doi:10.14336/AD.2022.0313)
Supplement: Supplementary file 1 — The Supplementary data can be found online at: www.aginganddisease.org/EN/10.14336/AD.2022.0313. [file AD-13-6-1939-s.pdf]

SUPPLEMENTARY DATA

Single-Cell RNA-Seq Reveals a Population of Smooth Muscle Cells Responsible for Atherogenesis

Xiaofeng Shi[1,2#], Shangming Zhu[1,3#], Meijing Liu[1], Sara Saymuah Stone[4], Yao Rong[1,3], Ke Mao[3], Xiaopeng Xu[1], Chao Ma[3], Zhuoyuan Jiang[3], Yan Zha[3], Chun Yan[1], Xiaofan Yu[3], Di Wu[5], Guiyou Liu[2], Jidong Mi[6], Jianping Zhao[6], Yuan Li[6], Yuchuan Ding[4], Xiaogang Wang[1,7*], Yong-Biao Zhang[1,7*], Xunming Ji[2,5*]

© 2022. Shi X et al. Published online at http://www.aginganddisease.org/EN/10.14336/AD.2022.0313

SUPPLEMENTARY DATA

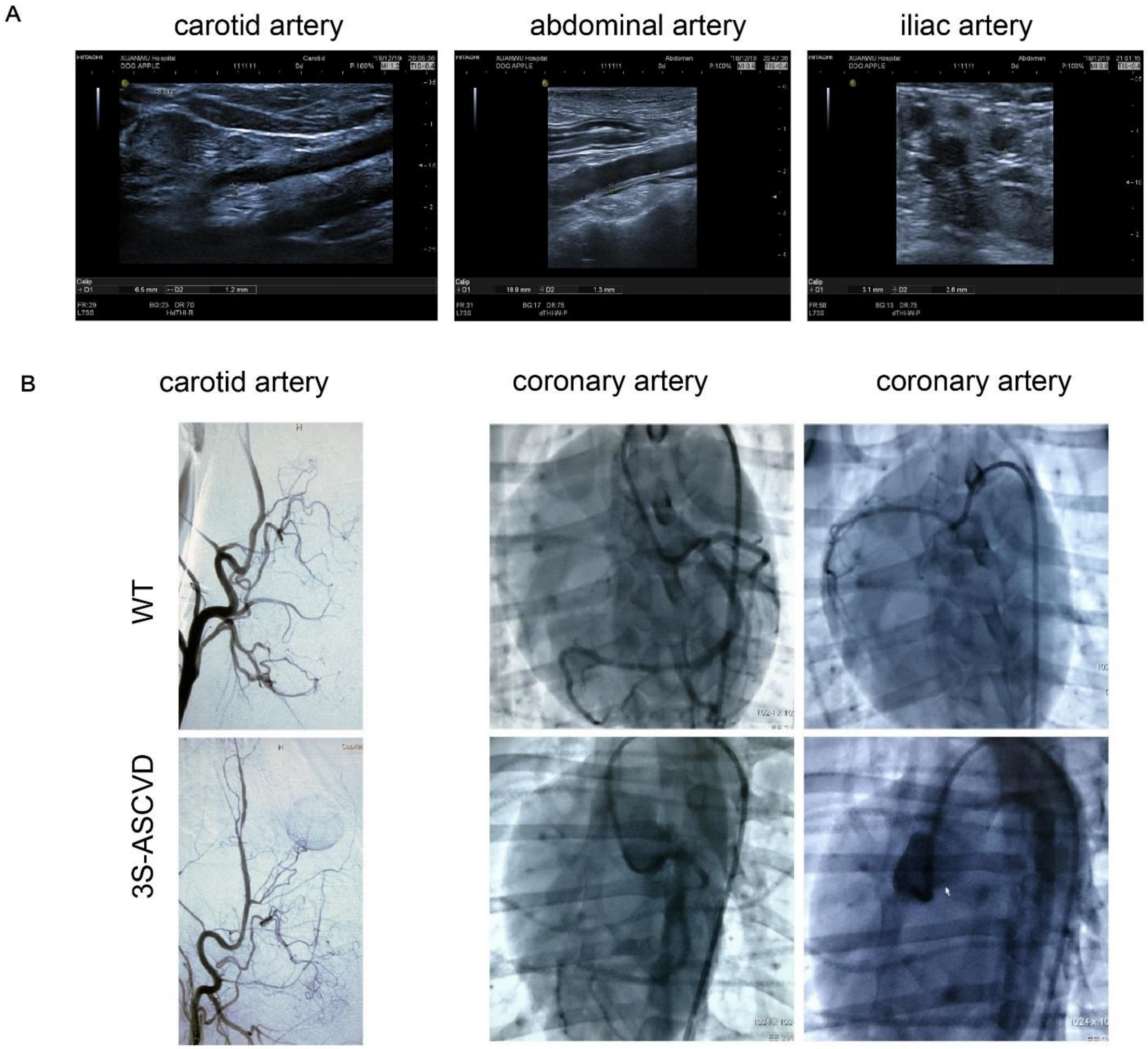

Supplementary Figure 1. Ultrasound and angiography features of the AS lesions in 3S-ASCVD dogs. (A) Ultrasound imaging of carotid, abdominal and iliac arteries. **(B)** Angiography imaging of carotid and coronary arteries.

© 2022. Shi X et al. Published online at http://www.aginganddisease.org/EN/10.14336/AD.2022.0313

SUPPLEMENTARY DATA

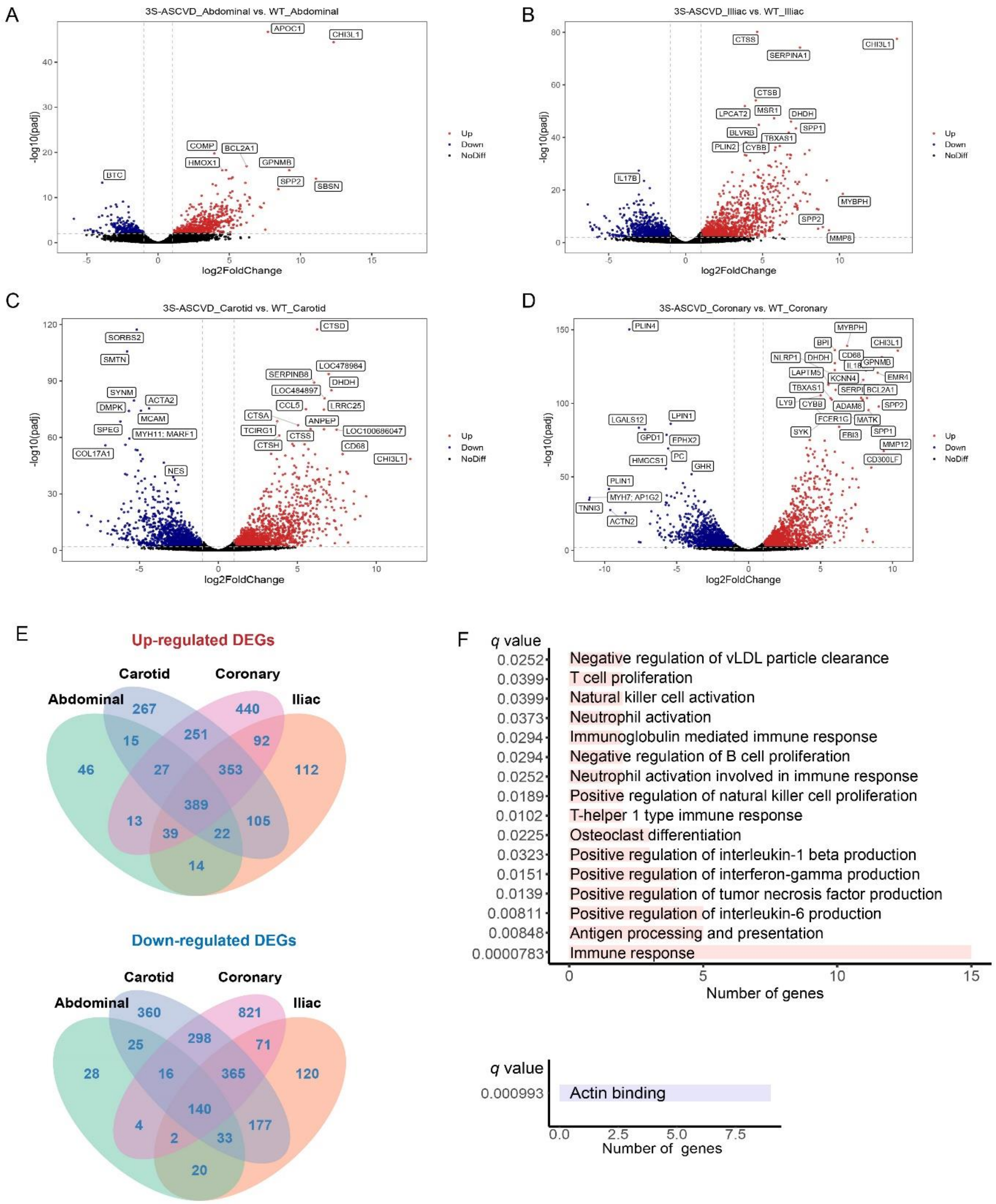

Supplementary Figure 2. Gene expression change of the four AS-prone arteries. (A-D) Volcano plots of the most significant DEGs (P<0.01, log_2(fold change)>3) in abdominal, iliac, carotid, coronary arteries, respectively. **(E)** Venn diagram analysis showed the common DEGs. **(F)** Significantly enriched GO terms of the common DEGs. Red, GO terms of up-regulated DEGs; blue, GO terms of down-regulated DEGs.

© 2022. Shi X et al. Published online at http://www.aginganddisease.org/EN/10.14336/AD.2022.0313

SUPPLEMENTARY DATA

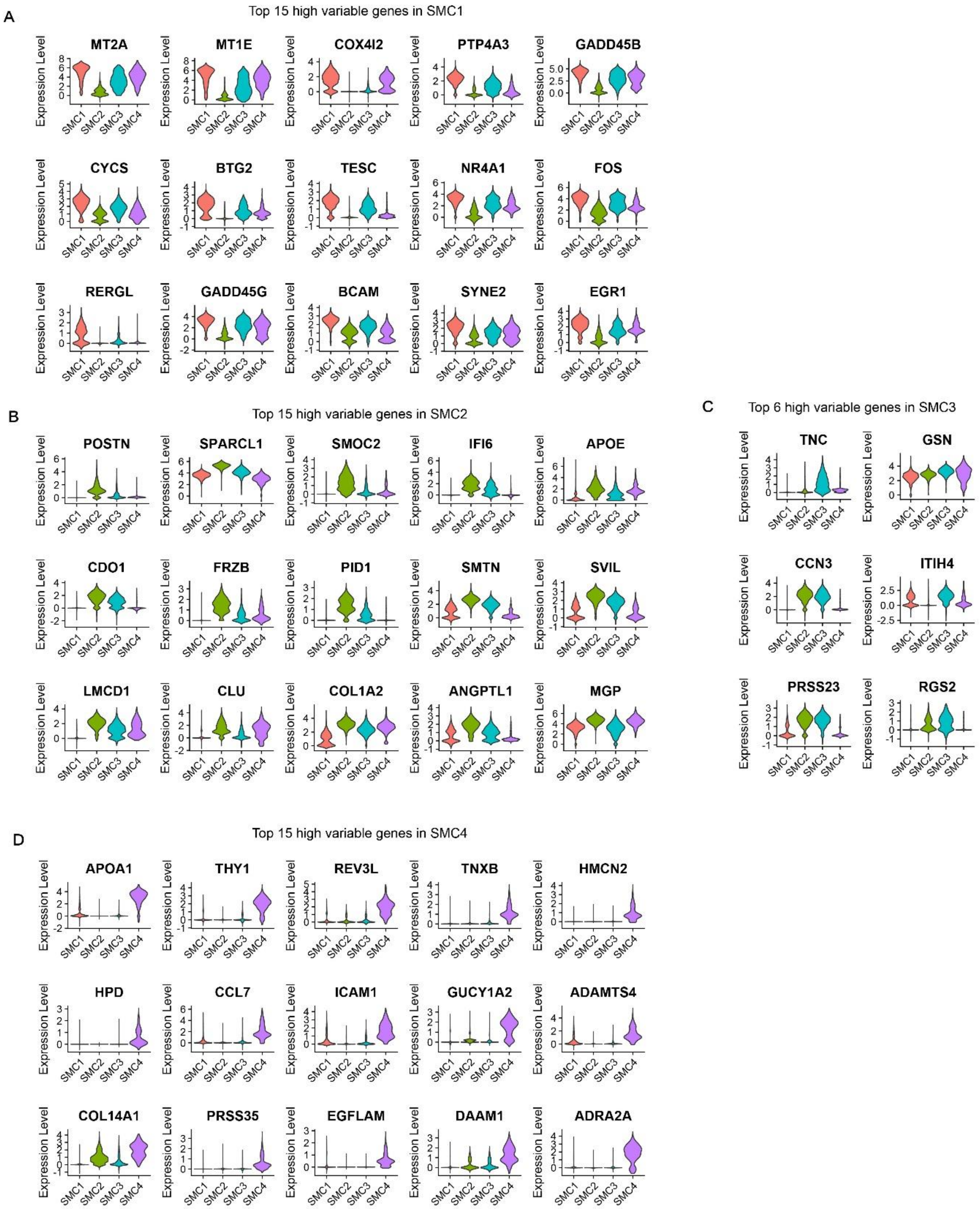

Supplementary Figure 3. Gene expression profiles of SMC cell subsets. (A-D) Violin plots showed the top highly variable genes in SMC1, SMC2, SMC3 and SMC4, respectively.

© 2022. Shi X et al. Published online at http://www.aginganddisease.org/EN/10.14336/AD.2022.0313

SUPPLEMENTARY DATA

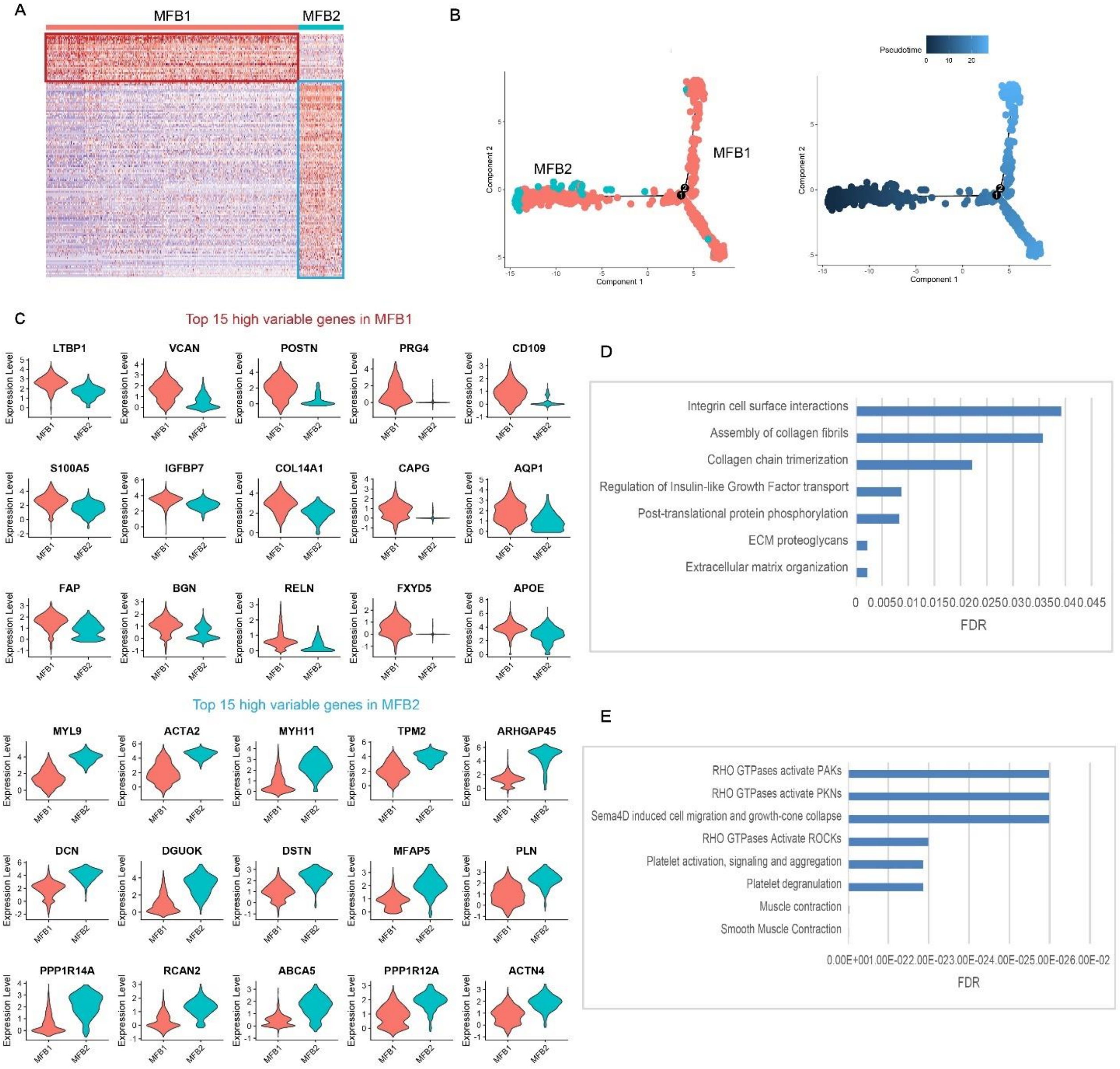

Supplementary Figure 4. Gene expression profiles of MFB cell subsets. (A) Heatmap of hierarchically clustered the highly variable genes across MFB cells (n= 1763 cells). (**B)** Pseudotime analysis showed the inter-cluster relationship of MFB cells. (**C)** Violin plots showed the top 15 highly variable genes in MFB1 and MFB2 cells, respectively. (**D, E)** GO enrichment analysis of the highly variable genes found in cells of MFB1 and MFB2 subsets, respectively.

© 2022. Shi X et al. Published online at http://www.aginganddisease.org/EN/10.14336/AD.2022.0313

SUPPLEMENTARY DATA

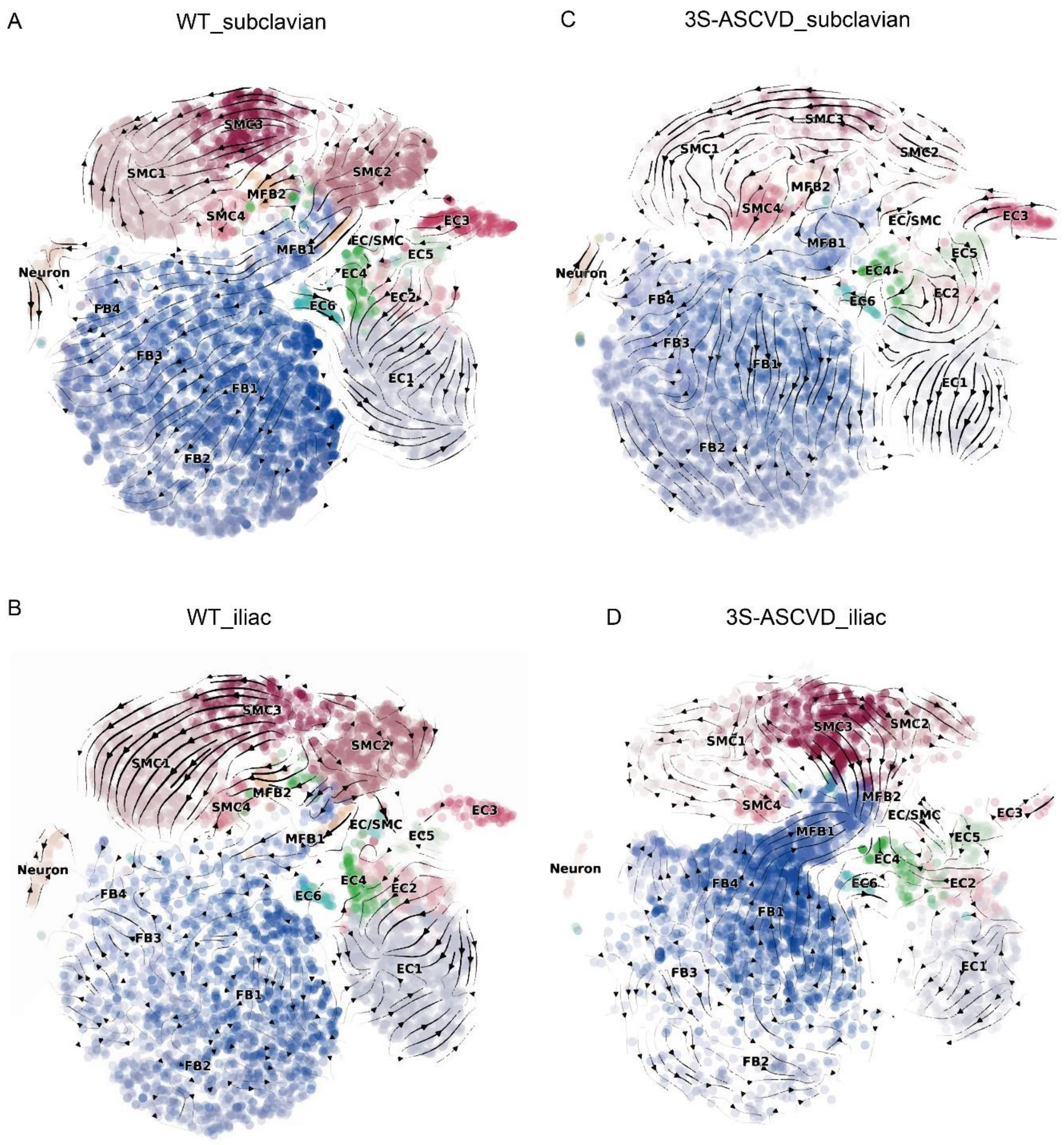

Supplementary Figure 5. RNA velocity analysis illustrates the extent and direction of cell differentiation in the t-SNE plot of AS-prone arteries and AS-resistant arteries. (A-D) Transcriptional dynamics of AS-prone arteries and AS-resistant arteries in WT and 3S-ASCVD dogs, respectively. The direction of arrow represents the future state of the cells. Velocity field superimposed to the t-SNE embedding of cells by sample. Cells are colored by cell type according to t-SNE uniform manifold approximation and projection.

© 2022. Shi X et al. Published online at http://www.aginganddisease.org/EN/10.14336/AD.2022.0313

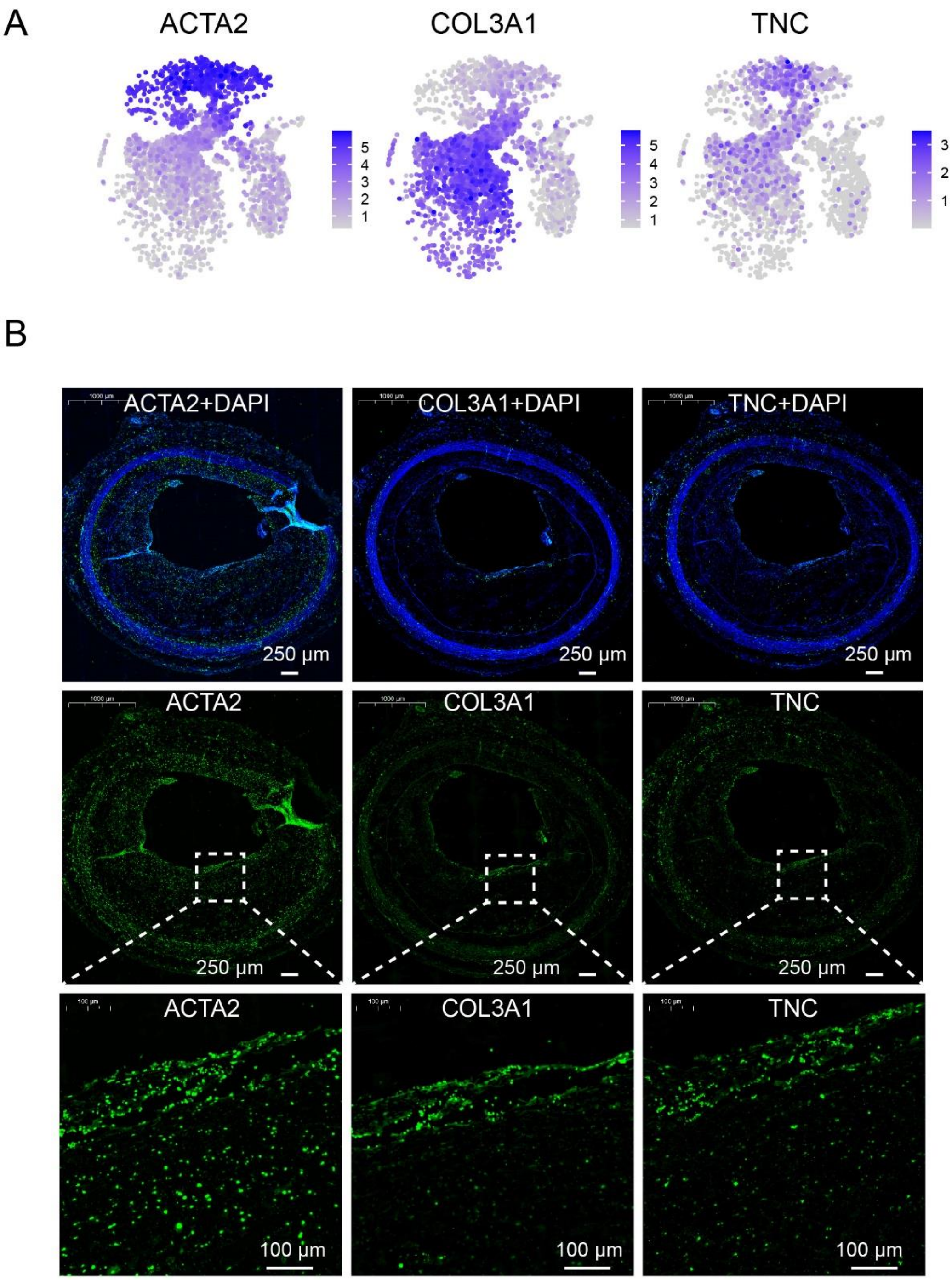

Supplementary Figure 6. Key genes detected by RNA-FISH. (A) The expression of ACTA2, COL3A1 and TNC in UMAP. (**B)** RNA-FISH of ACTA2, COL3A1 and TNC in AS-prone iliac arteries.

© 2022. Shi X et al. Published online at http://www.aginganddisease.org/EN/10.14336/AD.2022.0313

SUPPLEMENTARY DATA

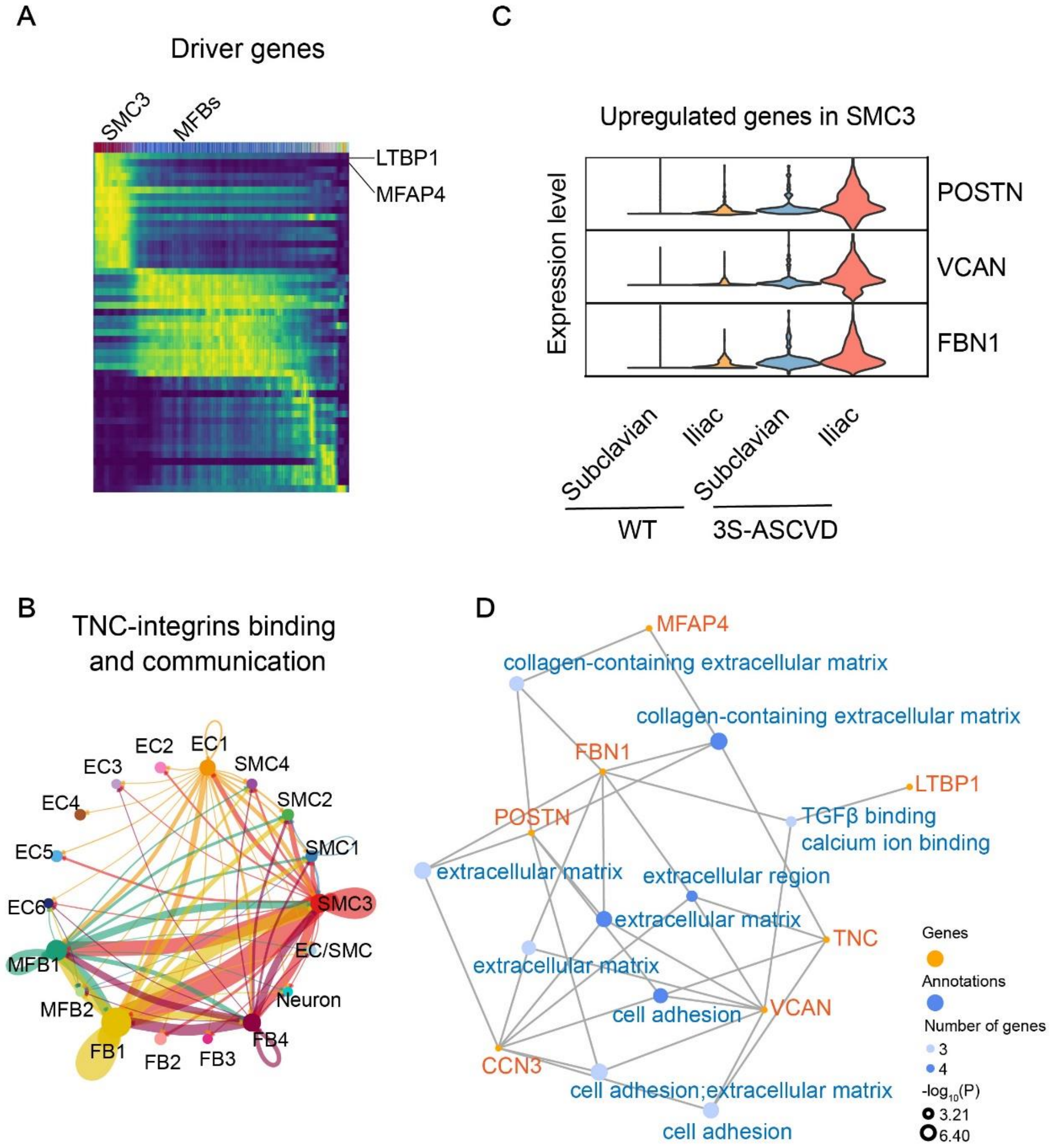

Supplementary Figure 7. TNC⁺ SMC-specific transcriptional changes. (A) Putative gene programs driving TNC⁺ SMC differentiation. (**B)** Circle plot showed the inferred tenascin signaling network. Significant ligand-receptor pairs sending signals from TNC⁺ SMC to MFB1, FB1 and FB4. (**C)** Violin plot showed the significantly DEGs in the cells of TNC⁺ SMC subset (3S-ASCVD versus WT). (**D)** Network of GO annotation terms for the key genes in cells of TNC⁺ SMC subset.

© 2022. Shi X et al. Published online at http://www.aginganddisease.org/EN/10.14336/AD.2022.0313

SUPPLEMENTARY DATA

Supplementary Table 1. Up- and down-regulated proteins in livers of 3S-ASCVD dogs.

Accession Number	Identified Proteins	***P***	Ratio
A0A5F4CSY2	Apolipoprotein E	0.001	0.193
E2QUN4	Aminoadipate aminotransferase	0.004	0.418
A0A5F4D8H1	Peptidylprolyl isomerase	0.005	0.458
A0A5F4CHR2	Myosin IE	0.028	0.467
KPYR	Pyruvate kinase PKLR	0.034	0.473
E2RM47	SEC14 like lipid binding 3	0.000	0.496
A0A0B4J196	Uncharacterized protein	0.000	0.517
E2RNZ3	Asparaginase	0.007	0.522
A0A5F4D7P7	Acyl-CoA thioesterase 12	0.004	0.524
F1PK43	Nuclear autoantigenic sperm protein	0.037	0.530
A0A5F4C5A6	Solute carrier organic anion transporter family member	0.005	0.534
E2RMX7	Aldedh domain-containing protein	0.029	0.536
A0A5F4C876	Maltase-glucoamylase	0.015	0.543
F1P976	Glycerol-3-phosphate dehydrogenase [NAD(+)]	0.001	0.543
E2RF91	Carboxymethylenebutenolidase homolog	0.004	0.544
A0A5F4C689	Lactate dehydrogenase D	0.025	0.550
E2RAD0	3-hydroxy-3-methylglutaryl coenzyme A synthase	0.004	0.560
E2QWA2	Aldose 1-epimerase	0.029	0.565
A0A5F4C4G5	Molybdenum cofactor synthesis 1	0.005	0.566
J9NU28	Lecithin retinol acyltransferase	0.034	0.583
A0A5F4C6T8	Sorting nexin 4	0.029	0.584
F1PSM2	Alpha-1,4 glucan phosphorylase	0.006	0.593
A0A5F4C7H6	Chromosome 21 C11orf54 homolog	0.045	0.594
A0A5F4CFX8	Alpha-1,4 glucan phosphorylase	0.002	0.595
A0A5F4BU84	Uncharacterized protein	0.026	0.603
A0A5F4CVN2	Hydroxyacyl-CoA dehydrogenase	0.005	0.605
A0A5F4BVG2	SEC14 like lipid binding 4	0.000	0.610
F6XUY6	Fructose-bisphosphate aldolase	0.007	0.613
F1PP35	Succinate-semialdehyde dehydrogenase	0.001	0.614
E2RN29	Uncharacterized protein	0.012	0.619
E2RLZ9	Abhydrolase domain containing 14B	0.005	0.619
E2RS63	Fumarylacetoacetase	0.002	0.621
A0A5F4CDG5	Sorting nexin 27	0.049	0.622
A0A5F4D3D6	Cytochrome P450 1A2	0.024	0.625
A0A5F4CCI2	Potassium voltage-gated channel interacting protein 3	0.003	0.629
DHDH	Trans-1,2-dihydrobenzene-1,2-diol dehydrogenase	0.008	0.645
A0A5F4CJ02	S-formylglutathione hydrolase	0.017	0.646
A0A5F4BZ81	EI24 autophagy associated transmembrane protein	0.014	0.650
A0A5F4D7A7	Kynureninase	0.008	0.650
A0A5F4CSH9	Carboxypeptidase Q	0.004	0.658
F1P9H9	Nuclear casein kinase and cyclin dependent kinase substrate 1	0.032	0.659
E2RNQ5	7-dehydrocholesterol reductase	0.010	0.659

© 2022. Shi X et al. Published online at http://www.aginganddisease.org/EN/10.14336/AD.2022.0313

J9NRX4	Uncharacterized protein	0.014	0.664
E2RHQ0	Aldehyde dehydrogenase 1 family member B1	0.040	0.668
F1PJP0	Ectonucleotide pyrophosphatase/phosphodiesterase 1	0.012	0.669
A0A5F4D5T7	Aldehyde dehydrogenase 7 family member A1	0.002	0.669
A0A5F4CMQ4	Glutathione S-transferase omega 1	0.001	0.673
E2RBM1	Ketohexokinase	0.009	0.674
E2QYQ4	Cytidine monophosphate N-acetylneuraminic acid synthetase	0.028	0.676
EPM2A	Laforin	0.002	0.677
A0A5F4C681	SAP domain containing ribonucleoprotein	0.038	0.680
F1P898	Hydrogen voltage gated channel 1	0.009	0.687
A0A5F4BWX0	RRM domain-containing protein	0.006	0.687
J9P7P2	Uncharacterized protein	0.040	0.689
A0A0A0MPD3	NAD(P)H-hydrate epimerase	0.000	0.692
E2RQ68	Ceramide synthase 2	0.043	0.693
F1PUL4	Phosphoglucomutase 1	0.003	0.693
A0A5F4BT61	Nipsnap homolog 3B	0.004	0.694
E2R125	Perilipin	0.046	0.694
A0A5F4DJH5	Cofilin 2	0.007	0.695
F1P6A6	Acyl-CoA synthetase medium chain family member 4	0.023	0.695
F1P9L1	S-methyl-5'-thioadenosine phosphorylase	0.031	0.696
A0A5F4CVL0	Phosphoglucomutase 1	0.001	0.696
F1PAB7	Aldehyde dehydrogenase 9 family member A1	0.046	0.700
A0A5F4DAM0	Solute carrier family 38 member 4	0.010	0.700
A0A5F4CV77	Coiled-coil domain containing 58	0.029	0.701
E2RCN0	Thiosulfate sulfurtransferase like domain containing 1	0.046	0.702
E2R0Y5	Succinate--CoA ligase [ADP/GDP-forming] subunit alpha, mitochondrial	0.017	0.705
F1PCF2	ATP binding cassette subfamily B member 11	0.001	0.707
F1PGI4	Starch binding domain 1	0.027	0.708
F6UT62	Sorting nexin 3	0.010	0.708
DLDH	Dihydrolipoyl dehydrogenase, mitochondrial	0.036	0.709
E2QV08	Malate dehydrogenase	0.018	0.711
F1PJS0	Glyoxylate and hydroxypyruvate reductase	0.002	0.711
F1PCS9	Uncharacterized protein	0.049	0.712
E2RRR2	Hydroxysteroid 17-beta dehydrogenase 14	0.002	0.712
A0A5F4D3F0	Dimethylaniline monooxygenase [N-oxide-forming]	0.014	0.713
A0A5F4CN31	Glucose-6-phosphate isomerase	0.004	0.716
A0A5F4C729	SEC23 interacting protein	0.032	0.719
A0A5F4D5L9	Hydroxylysine kinase	0.003	0.719
A0A5F4C4T7	Apoptotic chromatin condensation inducer 1	0.007	0.721
A0A5F4DI94	Isocitrate dehydrogenase [NAD] subunit, mitochondrial	0.023	0.721
F1PLW6	2'-5'-oligoadenylate synthetase 1	0.031	0.721
A0A5F4DBV6	Proline rich coiled-coil 1	0.042	0.723
E2R3I8	PKS_ER domain-containing protein	0.000	0.725
J9P8V6	Neutral cholesterol ester hydrolase 1	0.029	0.729
F1P6W6	Acyl-CoA dehydrogenase medium chain	0.016	0.729

© 2022. Shi X et al. Published online at http://www.aginganddisease.org/EN/10.14336/AD.2022.0313

E2RRC9	Phosphoglycerate kinase	0.024	0.729
F1PGS8	FAD-binding PCMH-type domain-containing protein	0.004	0.732
A0A5F4BUN7	Fructose-bisphosphatase 1	0.005	0.732
A0A5F4DDM1	MISP family member 3	0.006	0.735
E2QT84	Beta-ureidopropionase 1	0.026	0.739
J9P7Z1	Phenazine biosynthesis like protein domain containing	0.026	0.741
A0A5F4DDY1	Uncharacterized protein	0.033	0.742
A0A5F4DGS4	Argininosuccinate synthase 1	0.022	0.744
E2RT07	NADH:ubiquinone oxidoreductase subunit A6	0.005	0.744
E2RQ87	Nipsnap homolog 1	0.002	0.747
F1PT97	Actin-related protein 2/3 complex subunit	0.049	0.750
F1PYB6	3-hydroxyisobutyrate dehydrogenase	0.031	0.751
F1PBJ8	MAPK activated protein kinase 5	0.008	0.751
A0A5F4BPI8	StAR related lipid transfer domain containing 5	0.005	0.753
A0A5F4BUG8	Sorbin and SH3 domain containing 2	0.039	0.753
A0A5F4CH14	Phosphotriesterase related	0.026	0.754
A0A5F4CGV9	Ribosomal protein S6 kinase	0.014	0.759
A0A5F4DF94	Heparin binding growth factor	0.007	0.759
E2RH95	SH3 domain-binding glutamic acid-rich-like protein	0.004	0.763
E2R6L1	Tankyrase 1 binding protein 1	0.012	0.764
E2R7C2	Methylthioribose-1-phosphate isomerase	0.025	0.764
A0A5F4BP08	Transket_pyr domain-containing protein	0.047	0.769
E2R4L5	Choline dehydrogenase	0.034	0.769
E2RFD0	3-hydroxy-3-methylglutaryl-CoA lyase	0.003	0.771
A0A5F4BZ94	Erythrocyte membrane protein band 4.1 like 2	0.021	0.771
E2QXS7	Adenosylhomocysteinase	0.046	0.771
E2QV12	Phosphoglucomutase 2	0.009	0.772
A0A5F4CRP5	Acyl-CoA dehydrogenase short/branched chain	0.017	0.774
F1PZ34	Epidermal growth factor receptor pathway substrate 15 like 1	0.015	0.777
J9P147	Succinate dehydrogenase assembly factor 2, mitochondrial	0.019	0.778
J9PB80	Fms related tyrosine kinase 3	0.033	0.780
A0A5F4BVL8	Phytanoyl-CoA 2-hydroxylase	0.027	0.782
E2QX33	Coiled-coil and C2 domain containing 1A	0.040	0.782
E2R2G5	Peptidylprolyl isomerase	0.001	0.783
A0A5F4C0Q2	3'-phosphoadenosine 5'-phosphosulfate synthase 2	0.040	0.784
F1PTP2	Sepiapterin reductase	0.024	0.785
F1PTZ9	Glyceraldehyde-3-phosphate dehydrogenase	0.003	0.786
A0A5F4C0Z1	Coenzyme Q10B	0.025	0.787
A0A5F4DA78	Sulfotransferase	0.031	0.789
F1PXN3	Sialic acid acetylesterase	0.011	0.790
F1Q3S9	ATP-dependent 6-phosphofructokinase	0.026	0.790
E2R0P3	Isochorismatase domain containing 1	0.036	0.790
A0A5F4DKX0	Phenylalanyl-tRNA synthetase subunit alpha	0.036	0.790
A0A5F4DEZ3	Dihydropyrimidinase like 2	0.046	0.792
A0A5F4BR06	Ubiquitin like modifier activating enzyme 6	0.021	0.792
A0A5F4BR97	Cordon-bleu WH2 repeat protein like 1	0.025	0.793

© 2022. Shi X et al. Published online at http://www.aginganddisease.org/EN/10.14336/AD.2022.0313

A0A5F4D615	Enolase superfamily member 1	0.048	0.796
A0A5F4CTD1	ATP synthase subunit gamma	0.025	0.798
A0A5F4CRE2	Kynurenine aminotransferase 1	0.003	0.801
A0A5F4BQ51	Interleukin enhancer binding factor 3	0.035	0.801
E2RD27		0.014	0.802
F1PYG8	Malate dehydrogenase	0.038	0.803
F1P7M0	Aminopeptidase	0.019	0.803
E2RT23	Derlin	0.044	0.803
A0A5F4BWL8	Citrate lyase beta like	0.036	0.807
F1P6Y7	Tubulin polymerization promoting protein family member 3	0.008	0.809
A0A5F4CWN1	Xylulokinase	0.041	0.815
A0A5F4C7H9	Dihydropyrimidinase like 3	0.020	0.816
F1Q248	Solute carrier family 25 member 18	0.002	0.817
E2RL58	3-hydroxyisobutyryl-CoA hydrolase, mitochondrial	0.027	0.818
E2R0A7	Glycine-N-acyltransferase	0.041	0.819
H9GW61	Phosphotransferase	0.033	0.820
DHB8	Estradiol 17-beta-dehydrogenase 8	0.005	0.821
F1PFK5	Formimidoyltransferase cyclodeaminase	0.007	0.821
A0A5F4C8L8	NADH:ubiquinone oxidoreductase subunit A9	0.010	0.827
A0A5F4C5N2	Peroxiredoxin 6	0.047	0.828
A0A5F4CZK2	Rad60-SLD domain-containing protein	0.002	0.829
CP2E1	Cytochrome P450 2E1	0.007	1.201
A0A5F4BXY2	Calcium-transporting ATPase	0.015	1.203
A0A5F4D804	Voltage dependent anion channel 2	0.016	1.203
E2R4F5	60S ribosomal protein L13	0.010	1.204
A0A5F4CUN5	Kinectin 1	0.034	1.209
A0A5F4CDF0	Serine/arginine repetitive matrix 2	0.006	1.210
E2RHY7	Serpin family H member 1	0.020	1.210
RAN	GTP-binding nuclear protein Ran	0.032	1.210
A0A5F4CGS5	Fascin actin-bundling protein 1	0.009	1.211
E2RMT6	WD repeat domain 3	0.006	1.217
E2RM35	Pyrroline-5-carboxylate reductase	0.045	1.220
A0A5F4BZT3	ST6 beta-galactoside alpha-2,6-sialyltransferase 1	0.010	1.223
F2Z4N1	Uncharacterized protein	0.019	1.225
A0A5F4CVT3	MIC	0.013	1.229
A0A5F4CAA7	5'-nucleotidase domain containing 1	0.035	1.229
E2R9G7	Ribosomal protein L11	0.027	1.232
A0A5F4CWU1	Mitochondrial amidoxime reducing component 1	0.010	1.240
A0A5F4C006	Isochorismatase domain containing 2	0.047	1.242
A0A5F4CKH5	Hypoxia up-regulated 1	0.039	1.249
A0A5F4CT15	Protein phosphatase 1 regulatory subunit 7	0.026	1.250
A0A5F4BQW3	Dynactin subunit 2	0.009	1.251
A0A5F4DEW8	RL10P_insert domain-containing protein	0.024	1.252
A0A5F4DFY8	Splicing factor 3b subunit 3	0.007	1.254
E2RBM3	Uncharacterized protein	0.017	1.255
E2RQP6	Eukaryotic translation initiation factor 3 subunit C	0.016	1.257

F6UQZ9	Malonyl-CoA decarboxylase	0.015	1.257
A0A5F4C861	Dynamin 2	0.044	1.259
A0A5F4BQK3	Lysosomal associated membrane protein 2	0.030	1.259
A0A5F4CIY3	Signal recognition particle subunit SRP68	0.028	1.260
F1PML6	Enoyl-CoA delta isomerase 2	0.003	1.261
E2R0R1	Proteasome 26S subunit, non-ATPase 9	0.035	1.265
F1P843	ATP binding cassette subfamily D member 3	0.013	1.265
F6UZV8	Calpain 1	0.017	1.265
A0A5F4C4R0	Galectin	0.021	1.268
E2RR30	Fatty acid amide hydrolase	0.019	1.273
A0A5F4DBL7	LDL receptor related protein 1	0.014	1.273
F1P895	Uncharacterized protein	0.018	1.281
A0A5F4C2A9	Adducin 1	0.007	1.284
G1K268	Uncharacterized protein	0.012	1.285
F1P969	Eukaryotic translation initiation factor 3 subunit B	0.006	1.285
E2R043	UDP-glucuronosyltransferase	0.001	1.286
E2QW49	Proliferation-associated 2G4	0.009	1.286
E2QY43	Proteasome 26S subunit, non-ATPase 8	0.040	1.290
A0A5F4CGL0	Fe-S_biosyn domain-containing protein	0.009	1.290
A0A5F4DJ85	Rho GDP dissociation inhibitor alpha	0.010	1.290
A0A5F4BY07	Granzyme B	0.004	1.294
E2R548	Vimentin type intermediate filament associated coiled-coil protein	0.050	1.295
F1PD82	Terpene cyclase/mutase family member	0.047	1.297
F1PLN2	Uncharacterized protein	0.003	1.300
A0A5F4BTM5	Dihydroorotate dehydrogenase (quinone), mitochondrial	0.004	1.303
E2RB92	Mitochondrial ribosomal protein S5	0.009	1.303
A0A5F4CJ20	Integrin beta	0.008	1.304
A0A5F4CWA4	RAB29, member RAS oncogene family	0.015	1.305
A0A5F4CC58	Signal transducer and activator of transcription	0.004	1.306
E2R2N2	Mitogen-activated protein kinase	0.013	1.308
A0A5F4DAC9	Calcineurin like phosphoesterase domain containing 1	0.025	1.309
A0A5F4C7B0	Uncharacterized protein	0.041	1.310
E2RL34	60S ribosomal protein L18a	0.019	1.311
F1P909	NADH:ubiquinone oxidoreductase subunit A13	0.043	1.319
A0A5F4CCZ8	Pyridoxal dependent decarboxylase domain containing 1	0.043	1.321
A0A5F4C5K5	RAP1B, member of RAS oncogene family	0.005	1.321
F1PR81	Protein phosphatase 1 regulatory subunit	0.026	1.332
A0A5F4BUG3	Retinoid X receptor alpha	0.010	1.332
A0A5F4C9E5	60S ribosomal protein L12	0.041	1.333
E2RB06	VAC14 component of PIKFYVE complex	0.025	1.334
F2Z4P3	60S ribosomal protein L23	0.006	1.335
E2RK64	Fermitin family member 3	0.044	1.338
F1P7J7	Egl-9 family hypoxia inducible factor 1	0.032	1.340
A0A5F4CH29	StAR related lipid transfer domain containing 10	0.045	1.342
A0A5F4DDF5	Mannose-6-phosphate receptor, cation dependent	0.039	1.344
A0A5F4CLT5	NSF attachment protein alpha	0.006	1.364

© 2022. Shi X et al. Published online at http://www.aginganddisease.org/EN/10.14336/AD.2022.0313

A0A5F4BVZ3	Inter-alpha-trypsin inhibitor heavy chain 1	0.027	1.370
A0A5F4BV15	TBC1 domain family member 17	0.016	1.372
A0A5F4CV96	CAP-Gly domain containing linker protein 1	0.044	1.376
F1PSS8	Protein S	0.047	1.381
E2RJQ8	V-type proton ATPase subunit	0.012	1.391
F1PI88	UDP-glucose 4-epimerase	0.025	1.393
F1PGZ1	Beta-1,4-galactosyltransferase 1	0.044	1.394
E2R6I0	Bcl-2-like protein 2	0.009	1.397
F1PD80	Transcription factor BTF3	0.014	1.397
A0A5F4BS19	Tyrosine-protein phosphatase non-receptor type	0.013	1.403
E2QY42	Dynactin subunit 4	0.044	1.407
A0A5F4D0M8	Calpain 5	0.036	1.408
E2QZQ1	Transferrin	0.008	1.411
A0A5F4C665	SH3 domain containing GRB2 like, endophilin B2	0.008	1.422
E2QSF4	Tubulin beta chain	0.002	1.433
A0A5F4BNQ5	Switching B cell complex subunit SWAP70	0.036	1.441
J9NU66	Acid phosphatase 1	0.039	1.447
E2RKJ6	Serpin family B member 1	0.041	1.453
A0A5F4C9D6	Flotillin	0.003	1.458
A0A5F4CBF1	Uncharacterized protein	0.023	1.461
A0A0A0MPC0	Translocon-associated protein subunit alpha	0.018	1.470
A0A5F4C6Z8	Ribos_L4_asso_C domain-containing protein	0.005	1.477
A0A5F4BUQ6	Phospholipid-transporting ATPase	0.003	1.481
A0A5F4DCY9	Ig-like domain-containing protein	0.004	1.484
A0A5F4CYE8	DnaJ heat shock protein family (Hsp40) member B1	0.029	1.484
E2RF42	Amidophosphoribosyltransferase	0.037	1.485
F1PFR1	RAB33B, member RAS oncogene family	0.042	1.492
A0A5F4C1K4	2',3'-cyclic nucleotide 3' phosphodiesterase	0.023	1.510
A0A5F4CVB7	Prostaglandin F2 receptor inhibitor	0.017	1.510
E2R0M4	N-acetylglucosamine-1-phosphate transferase subunit gamma	0.032	1.514
E2R1S5	Eukaryotic translation initiation factor 6	0.013	1.519
E2RKV3	Serine palmitoyltransferase long chain base subunit 1	0.020	1.519
F1PAG7	LDL receptor related protein associated protein 1	0.045	1.521
A0A5F4BRW9	Thrombospondin 1	0.013	1.523
E2RL97	Canopy FGF signaling regulator 4	0.035	1.523
E2QS21	Family with sequence similarity 98 member B	0.019	1.525
E2RJG1	Armadillo repeat containing X-linked 3	0.019	1.532
E2RA06	KN motif and ankyrin repeat domains 2	0.044	1.534
A0A5S6DEE6	Clusterin	0.002	1.535
A0A5F4CTD0	Alpha-1-B glycoprotein	0.019	1.537
A0A5F4D5S2	Uncharacterized protein	0.029	1.541
A0A5F4CQ34	Proline dehydrogenase	0.046	1.544
F1PLS0	Sec1 family domain containing 1	0.041	1.548
A0A5F4DCK8	Farnesyl-diphosphate farnesyltransferase 1	0.004	1.552
A0A5F4DB26	Coagulation factor IX	0.041	1.558
A0A5F4CAL0	Complement C7	0.009	1.569

A0A5F4DHB9	Ribosomal protein S13	0.014	1.571
A0A5F4BNM9	Galectin	0.004	1.574
J9PAG9	Transmembrane protein 126A	0.043	1.583
E2RC86	40S ribosomal protein S30	0.009	1.586
A0A5F4CKM6	Uncharacterized protein	0.012	1.595
F1PAL5	Angiotensinogen	0.036	1.596
A0A5F4D8C0	Golgi transport 1B	0.024	1.596
A0A5S6DQF4	Ferritin	0.015	1.615
A0A5F4DI16	Hydroxyacid oxidase 2	0.009	1.628
A0A5F4BUW5	Retinol dehydrogenase 13	0.002	1.646
A0A5F4C930	Kinesin family member 1C	0.018	1.651
A0A5F4BUZ4	Hepsin	0.041	1.651
F1PB10	Y-box binding protein 3	0.001	1.653
A0A5F4BV87	Tubulin tyrosine ligase like 12	0.009	1.661
E2RPU3	Ubiquitination factor E4A	0.022	1.673
F1PCE5	Serpin family A member 1	0.003	1.676
A0A5F4CNP2	UDPGT domain-containing protein	0.026	1.678
F1PMP8	Non-specific protein-tyrosine kinase	0.001	1.679
A0A5F4CQI6	Alpha-mannosidase	0.020	1.682
F1PG16	Uncharacterized protein	0.017	1.687
A0A5F4C1Z1	Ribosomal_L7Ae domain-containing protein	0.004	1.689
F1P8Z5	Apolipoprotein B	0.008	1.690
E2RLB3	Peptidylprolyl isomerase	0.001	1.691
F1PH87	Serpin family A member 3	0.030	1.703
J9P028	Glutathione peroxidase	0.010	1.715
A0A5F4DA28	Hypoxanthine phosphoribosyltransferase	0.009	1.722
C5AR1	C5a anaphylatoxin chemotactic receptor 1	0.028	1.734
A0A5F4DHS3	Intercellular adhesion molecule 3	0.006	1.760
A0A5F4BQ64	Uncharacterized protein	0.003	1.770
A0A5F4CA49	Kininogen 1	0.022	1.770
A0A5F4BZM5	Ceruloplasmin	0.004	1.810
F1Q421	Plasminogen	0.005	1.815
F1P9G4	DEK proto-oncogene	0.036	1.820
APOC2	Apolipoprotein C-II	0.014	1.846
A0A5F4CR45	Complement C2	0.027	1.849
F1PGM1	Complement C3	0.003	1.863
E2RDW4	Lectin, mannose binding 2 like	0.008	1.867
J9P7F7	Ficolin 1	0.033	1.872
E2R141	Complement C8 beta chain	0.001	1.889
E2RFV9	Complement C9	0.013	1.942
A0A5F4C561	Ferritin	0.001	1.966
A0A5F4BYJ5	Uncharacterized protein	0.043	2.014
A0A5S6CZL3	Apolipoprotein A-IV	0.039	2.079
F1P7J4	Complement C5	0.002	2.090
E2R0V0	BCL2 like 13	0.046	2.095
A0A5F4CE30	Ig-like domain-containing protein	0.002	2.132

© 2022. Shi X et al. Published online at http://www.aginganddisease.org/EN/10.14336/AD.2022.0313

B4YY02	Adenylate kinase isoenzyme 1	0.030	2.136
E2R1N1	Y	0.008	2.157
F1PDY8	Carbonic anhydrase 2	0.018	2.181
A0A5F4D598	Ubiquitin carboxyl-terminal hydrolase	0.005	2.187
A0A5F4CNZ1	Complement C6	0.004	2.206
A0A5F4CVJ2	Ig-like domain-containing protein	0.008	2.228
E2RGI4	Chloride intracellular channel protein	0.030	2.228
A0A5F4BRF7	Bridging integrator 1	0.012	2.248
E2QVV7	DNA polymerase delta interacting protein 2	0.045	2.265
F1PZR4	Amyloid beta precursor protein binding family B member 1	0.004	2.266
A0A5F4DFF1	Uncharacterized protein	0.011	2.275
F1PCG4	Peroxiredoxin 2	0.016	2.293
A0A5F4CZQ4	Non-specific serine/threonine protein kinase	0.031	2.331
J9PAQ5	Protein S100	0.010	2.467
A0A5F4C8F3	Ankyrin 1	0.035	2.519
A0A5F4BVF9	Beta-2-glycoprotein 1	0.029	2.529
A0A5F4CBH6	Ig-like domain-containing protein	0.044	2.552
E2RS75	Uncharacterized protein	0.001	2.603
F2Z4N3	60S ribosomal protein L27	0.011	2.633
F1PBL4	Fibrinogen alpha chain	0.047	2.737
E2R081	Serpin family B member 10	0.032	2.777
F1Q253	Spectrin beta chain	0.034	2.808
A0A5F4BWK7	Histone H2B	0.019	2.880
G1K2D9	Haptoglobin	0.009	2.900
HBB	Hemoglobin subunit beta	0.023	2.924
J9JHF7	GL	0.031	3.062
E2RSX8	Latexin	0.007	3.159
A0A5F4CXD8	Uncharacterized protein	0.001	3.212
F1PYZ1	Uncharacterized protein	0.030	3.213
E2R5J0	Pentaxin	0.046	3.353
H9GWY3	Inter-alpha-trypsin inhibitor heavy chain 4	0.005	3.765
A0A5F4CYK9	Dihydrolipoyl dehydrogenase	0.010	3.920
A0A5F4DEN8	RPL17-C18orf32 readthrough	0.032	3.971
APOA4	Apolipoprotein A-IV	0.019	4.191
E2R833	Leucine rich alpha-2-glycoprotein 1	0.003	4.508
E2QYX1	Phosphoglycerate mutase	0.026	5.217

Note: Applicable data are numbers of arteries with percentages in parentheses. The imaging features without any of the specific findings including intramural hematoma (IMH), intimal flap, and double lumen are non-specific findings.

SUPPLEMENTARY DATA

Supplementary Table 2. Top ten up and down DEGs in 3S-ASCVD dogs.

Gene Symbol	log_2(fold change)	*P*	adjusted *P*	up/down
**Abdominal arteries**				
CHI3L1	12.33	5.45E-49	3.76E-45	Up
SBSN	11.09	6.04E-18	6.42E-15	Up
GPNMB	9.21	6.04E-20	9.27E-17	Up
SPP2	8.44	2.14E-15	1.48E-12	Up
APOC1	7.71	1.38E-51	1.91E-47	Up
CD300LF	7.53	5.45E-05	1.32E-03	Up
ALOX15B	7.43	5.02E-12	1.83E-09	Up
FGG	7.25	8.52E-11	1.76E-08	Up
CYP2S1	7.16	5.33E-11	1.17E-08	Up
SHC3	6.67	9.93E-09	1.10E-06	Up
MEI4	-5.91	5.85E-08	5.02E-06	down
ACTC1	-5.13	8.78E-05	1.96E-03	down
SH3GL2	-4.94	7.81E-05	1.79E-03	down
RIMS3	-4.75	4.16E-05	1.07E-03	down
SYT1	-4.60	1.20E-04	2.49E-03	down
MYH15	-4.59	5.27E-04	7.93E-03	down
CCDC42	-4.55	2.42E-04	4.26E-03	down
SLC4A5	-4.51	1.12E-04	2.35E-03	down
GJA5	-4.31	1.12E-04	2.36E-03	down
TMEM151B	-4.22	3.75E-04	6.12E-03	down
**Iliac arteries**				
CHI3L1	13.72	4.39E-82	3.14E-78	Up
MYBPH	10.21	2.09E-21	2.65E-19	Up
MMP8	9.32	1.39E-06	2.00E-05	Up
GPNMB	9.17	9.45E-27	2.15E-24	Up
SPP2	8.92	5.42E-08	1.10E-06	Up
LTF	8.63	3.84E-15	2.23E-13	Up
MMP9	8.61	3.06E-07	5.19E-06	Up
CD300LF	8.53	2.52E-15	1.51E-13	Up
MARCO	8.08	3.35E-21	4.07E-19	Up
EMR4	8.05	9.13E-39	6.35E-36	Up
COL17A1	-6.35	3.56E-17	2.84E-15	down
THNSL2	-6.22	7.15E-10	2.04E-08	down
AK8	-6.18	3.25E-08	6.96E-07	down
FBXO40	-6.05	2.93E-07	4.98E-06	down
SYT1	-6.05	1.32E-06	1.92E-05	down
SOST	-6.05	6.98E-07	1.09E-05	down
MEI4	-5.92	7.94E-09	1.89E-07	down
BTC	-5.80	5.75E-08	1.16E-06	down
CNTN1	-5.73	1.18E-03	7.29E-03	down
GCGR	-5.63	1.67E-07	3.01E-06	down

© 2022. Shi X et al. Published online at http://www.aginganddisease.org/EN/10.14336/AD.2022.0313

SUPPLEMENTARY DATA

**Carotid arteries**				
CHI3L1	12.16	7.57E-52	2.61E-49	Up
HS3ST2	9.36	2.09E-31	2.03E-29	Up
SPOCD1	8.98	1.26E-20	5.50E-19	Up
EMR4	8.65	8.48E-19	3.02E-17	Up
GPNMB	8.60	4.27E-34	4.76E-32	Up
CASP14	8.57	2.53E-27	1.79E-25	Up
NAT14	8.22	9.89E-06	7.27E-05	Up
EBI3	8.19	8.43E-44	1.92E-41	Up
CYP2S1	8.11	7.92E-45	1.93E-42	Up
HEPH	8.09	6.01E-23	3.13E-21	Up
CRISPLD1	-8.85	9.31E-23	4.79E-21	down
COL4A6	-8.29	1.32E-15	3.54E-14	down
EPYC	-8.04	1.36E-20	5.89E-19	down
COL6A6	-7.47	3.13E-12	5.76E-11	down
MYO18B	-7.46	1.47E-17	4.64E-16	down
COL17A1	-7.15	3.01E-59	1.58E-56	down
CARNS1	-7.01	7.17E-11	1.13E-09	down
CNTN1	-6.98	6.47E-18	2.12E-16	down
ADRA1B	-6.78	2.48E-10	3.68E-09	down
SH3RF2	-6.71	4.79E-13	9.75E-12	down
**Coronary arteries**				
CHI3L1	10.37	4.22E-140	1.34E-136	Up
MMP12	9.38	1.09E-70	3.02E-68	Up
GPNMB	9.26	1.83E-135	3.88E-132	Up
SPP2	9.04	2.84E-101	1.57E-98	Up
EMR4	8.97	1.52E-124	1.93E-121	Up
OSM	8.64	2.62E-07	1.95E-06	Up
CD300LF	8.52	2.24E-59	4.53E-57	Up
SPP1	8.34	9.83E-99	5.01E-96	Up
CCR5	8.25	1.10E-14	2.07E-13	Up
BCL2A1	8.23	3.57E-107	2.39E-104	Up
TNNI3	-11.08	4.23E-37	3.26E-35	down
MYH7; AP1G2	-11.04	1.42E-38	1.16E-36	down
PLIN1	-9.70	1.84E-44	1.94E-42	down
ACTN2	-9.60	6.03E-30	3.24E-28	down
TNNT2	-8.56	5.16E-28	2.54E-26	down
PLIN4	-8.29	4.13E-155	5.26E-151	down
LGALS12	-7.62	1.21E-86	4.40E-84	down
MYLK3	-7.62	2.40E-07	1.80E-06	down
CMYA5	-7.51	4.68E-07	3.31E-06	down
GPD1	-7.20	1.69E-85	5.80E-83	down

Note: Applicable data are numbers of arteries with percentages in parentheses.

© 2022. Shi X et al. Published online at http://www.aginganddisease.org/EN/10.14336/AD.2022.0313

SUPPLEMENTARY DATA

Supplementary Table 3. List of DEGs in AS-prone arteries in WT beagle dogs.

Gene Symbol	Log_2(fold change)	*P*	Adjusted *P*	Up/Down
P2RX1	4.838354306	7.23E-12	3.60E-08	Up
COL8A1	3.763732094	2.85E-11	1.07E-07	Up
MYL4	3.248770886	4.53E-06	0.0017816	Up
LDB3	2.97874036	2.97E-08	2.96E-05	Up
IGFBP2	2.949945423	8.31E-15	6.21E-11	Up
SHROOM3	2.853564457	6.42E-09	8.72E-06	Up
FBXO34	2.840277718	2.95E-05	0.006422	Up
TNC	2.731994213	1.92E-08	2.05E-05	Up
CARNS1	2.719096713	1.72E-09	4.28E-06	Up
COL17A1	2.666902928	2.23E-07	0.0001518	Up
TRPC3	2.618487539	5.26E-09	7.86E-06	Up
CHRNA6	2.616622335	6.11E-07	0.0003512	Up
TGFB3	2.411868951	1.19E-07	9.39E-05	Up
P2RY14	2.376445587	2.11E-05	0.0049364	Up
INSC	2.313702723	4.04E-05	0.0078486	Up
RAPSN	2.245312672	1.05E-07	8.73E-05	Up
RGS6	2.212992853	3.24E-05	0.0066614	Up
SLC6A17	1.949096773	8.85E-08	7.79E-05	Up
SOX6	1.931600718	1.18E-08	1.47E-05	Up
COL11A1	1.840406633	1.40E-05	0.0038097	Up
TRIM36	1.755199734	1.32E-06	0.0006231	Up
SPTLC3	1.665686103	9.87E-06	0.0028927	Up
DOCK10	1.585201625	2.06E-06	0.0009317	Up
SERPINI1	1.582918341	1.10E-05	0.0031624	Up
TMEM51	1.430843688	5.91E-08	5.53E-05	Up
KLHL38	1.349975778	3.14E-06	0.0013819	Up
PRDM11	1.29168392	1.39E-08	1.59E-05	Up
PHTF2	1.222636838	5.16E-06	0.001881	Up
SYDE2	1.210092795	1.12E-06	0.0005765	Up
PDE4D	1.159419488	4.49E-07	0.0002797	Up
IL15RA	1.129535281	3.88E-05	0.0076367	Up
PHLDB2	1.11311951	2.45E-09	4.76E-06	Up
DNAJB4	1.108983025	5.55E-05	0.0099995	Up
SMC4	1.077178168	6.70E-07	0.000371	Up
ECE1	1.059879623	6.52E-06	0.0021671	Up
KLHL30	1.051391603	5.04E-06	0.001881	Up
CCDC56	-7.519661714	2.55E-09	4.76E-06	down
LOC611363	-3.769023414	6.10E-06	0.0020717	down
TSPAN8	-3.175992114	1.33E-06	0.0006231	down
SHC4	-3.02667351	4.15E-09	6.90E-06	down

© 2022. Shi X et al. Published online at http://www.aginganddisease.org/EN/10.14336/AD.2022.0313

NDUFA4L2	-3.02182903	1.72E-07	0.0001228	down
SLC4A5	-2.926198304	2.53E-05	0.0058173	down
FBXL16	-2.839494713	1.68E-05	0.0042672	down
MDGA2	-2.62429086	5.02E-06	0.001881	down
CA9	-2.34805927	1.38E-05	0.0038097	down
NCAM1	-1.766946934	2.91E-05	0.006422	down
FSD1	-1.706198338	3.73E-06	0.0015825	down
FBP2	-1.705493887	8.88E-07	0.000474	down
RIC3	-1.5808313	1.75E-05	0.0043524	down
ACHE	-1.484121313	5.40E-05	0.0098546	down
HOXB4	-1.420236618	3.92E-06	0.0015825	down
PRXL2B	-1.319447473	2.96E-05	0.006422	down
PPP2R2B	-1.25634231	8.25E-06	0.002681	down
SLC2A1	-1.216958278	1.52E-05	0.0039949	down
KIAA1217	-1.187045406	4.61E-05	0.0086147	down
TRIM46	-1.180304972	1.79E-05	0.0043781	down
GRAMD1B	-1.099614477	5.31E-05	0.0098035	down

Supplementary Table 4. Summary for the scRNA-seq data of beagle dogs.

Samples	Cell Number	Mean Reads per Cell	Median Genes per Cell	Valid Barcodes	Sequencing Saturation	Reads Mapped to Genome	Total Genes Detected
WT_subclavian	9386	58055	1346	0.982	0.763	0.798	14944
WT_iliac	7412	75655	1345	0.982	0.815	0.798	14686
3S-ASCVD_subclavian	9759	66335	1206	0.971	0.796	0.848	15634
3S-ASCVD_iliac	6902	86029	1714	0.962	0.75	0.792	15161

Supplementary Table 5. List of highly variable genes for the four SMC subsets in beagle dogs.

Gene	Log_2(fold change)	Adjusted *P*	Cluster
ENSCAFG00000009113	1.654001771	0	SMC1
MT2A	1.644799526	0	SMC1
MT1E	1.544761997	0	SMC1
COX4I2	1.426057364	0	SMC1
PTP4A3	1.405431132	0	SMC1
GADD45B	1.209129748	0	SMC1
ENSCAFG00000028930	1.196764453	0	SMC1
CYCS	1.165020371	0	SMC1
BTG2	1.117519462	0	SMC1
TESC	1.093249744	0	SMC1
NR4A1	1.068874807	0	SMC1
FOS	1.052806476	0	SMC1
RERGL	1.03118663	0	SMC1

© 2022. Shi X et al. Published online at http://www.aginganddisease.org/EN/10.14336/AD.2022.0313

GADD45G	1.005108575	0	SMC1
BCAM	0.994502119	0	SMC1
SYNE2	0.949596535	0	SMC1
EGR1	0.923730844	0	SMC1
MGLL	0.88290045	0	SMC1
UBA2	0.867724608	0	SMC1
CRIP1	0.830257347	0	SMC1
RAMP1	0.793295421	0	SMC1
FTH1.1	0.7556921	0	SMC1
MT-CO1	0.698683142	0	SMC1
PPP1R12A	0.543618058	0	SMC1
COX6A2	0.95059245	1.16E-302	SMC1
PPP1R15A	0.781770294	2.12E-294	SMC1
ITIH5	0.581682052	1.74E-279	SMC1
ATF3	0.81338654	4.47E-262	SMC1
ARHGAP29	0.760340685	2.50E-249	SMC1
TIPARP	0.802704625	1.16E-246	SMC1
FOSB	0.682039121	1.48E-232	SMC1
CRIP2	0.608926237	3.99E-231	SMC1
EPHX1	0.775470166	9.83E-231	SMC1
SLC25A4	0.628076559	1.30E-230	SMC1
ZFP36	0.675813583	1.21E-215	SMC1
RHOB	0.723567094	5.01E-210	SMC1
GPRC5C	0.838610742	9.47E-199	SMC1
ENSCAFG00000008661	0.873702014	1.23E-197	SMC1
FOSL2	0.704923317	5.07E-195	SMC1
NR4A2	0.6560597	1.25E-194	SMC1
ARL5B	0.773711465	4.24E-189	SMC1
ID1	0.871124325	2.28E-185	SMC1
MAL	0.72732779	1.69E-174	SMC1
APOLD1	0.616149079	3.92E-172	SMC1
GLRX	0.74371725	4.88E-170	SMC1
NTRK2	0.59688202	1.03E-169	SMC1
IRF1	0.639322041	2.55E-169	SMC1
ITGA7	0.514022561	2.49E-157	SMC1
PGF	0.585024077	3.54E-156	SMC1
UTRN	0.722810591	8.39E-154	SMC1
AKAP12	0.776318848	1.11E-153	SMC1
ITIH1	0.737695054	3.29E-152	SMC1
MAFB	0.651874924	5.05E-149	SMC1
GADD45A	0.731816714	5.18E-145	SMC1
UBE2S	0.615493086	5.49E-136	SMC1
TRIB1	0.630296746	8.41E-135	SMC1
HERPUD1	0.640749426	3.05E-130	SMC1
ENSCAFG00000031852	0.563050699	5.84E-130	SMC1

© 2022. Shi X et al. Published online at http://www.aginganddisease.org/EN/10.14336/AD.2022.0313

SLC2A3	0.527735391	1.36E-129	SMC1
CFI	0.599758518	5.73E-122	SMC1
DNAJA1	0.510819781	1.02E-120	SMC1
SLC30A1	1.165045844	8.43E-120	SMC1
NRGN	0.530953075	3.87E-111	SMC1
GTF2B	0.519369616	1.28E-109	SMC1
NMU	0.579107535	1.49E-109	SMC1
PDE6H	1.07495163	1.93E-107	SMC1
SERTAD3	0.502137454	2.12E-105	SMC1
IFRD1	0.505021307	4.12E-104	SMC1
SULT1A1	0.587295886	4.42E-96	SMC1
THBS1	0.536223777	5.40E-96	SMC1
ZNF593	0.635615798	4.02E-92	SMC1
RASL11A	0.617134083	3.28E-88	SMC1
TNFRSF6B	0.561661142	1.04E-48	SMC1
IL6	0.647472091	6.84E-09	SMC1
POSTN	1.491734054	0	SMC2
SPARCL1	1.396648564	0	SMC2
SMOC2	1.380085741	0	SMC2
IFI6	1.379087512	0	SMC2
APOE	1.336005359	0	SMC2
CDO1	1.29989898	0	SMC2
FRZB	1.271844371	0	SMC2
PID1	1.261191391	0	SMC2
SMTN	1.233336251	0	SMC2
SVIL	1.164842165	0	SMC2
LMCD1	1.155032759	0	SMC2
CLU	1.134624965	0	SMC2
COL1A2	1.041757993	0	SMC2
ANGPTL1	1.036340535	0	SMC2
MGP	1.032217053	0	SMC2
CCN3	0.998810335	0	SMC2
SQOR	0.979271485	0	SMC2
DGUOK	0.975741215	0	SMC2
PRUNE2	0.920774986	0	SMC2
ENSCAFG00000028765	0.915149446	0	SMC2
TMEM45A	0.904671681	0	SMC2
FBN1	0.870292804	0	SMC2
NEXN	0.845518185	0	SMC2
PPP1R14A	0.809451763	0	SMC2
ENSCAFG00000005768	0.79819902	0	SMC2
VCAN	0.76677056	0	SMC2
PCSK1N	0.745526323	0	SMC2
MYOZ2	0.7242122	0	SMC2
FAP	0.70839358	0	SMC2

© 2022. Shi X et al. Published online at http://www.aginganddisease.org/EN/10.14336/AD.2022.0313

ILK	0.657398725	0	SMC2
NCAM1	0.642895051	0	SMC2
EDIL3	0.626420714	0	SMC2
MAOB	0.610406539	0	SMC2
RGS5	0.599276429	0	SMC2
PCDH7	0.557024961	0	SMC2
PTGS1	0.501287108	3.46E-300	SMC2
PDLIM2	0.765364092	8.20E-297	SMC2
PCBD1	0.758623885	1.30E-288	SMC2
ENSCAFG00000024781	0.698829205	2.45E-282	SMC2
TTLL7	0.720227009	1.38E-262	SMC2
ENSCAFG00000011915	0.734259009	1.78E-257	SMC2
MYH10	0.975574555	1.13E-251	SMC2
HSPB1	0.532961861	3.25E-251	SMC2
ENSCAFG00000045858	0.711538559	2.51E-250	SMC2
MAP1B	0.660397056	2.43E-240	SMC2
ENSCAFG00000023111	0.616282653	3.82E-231	SMC2
CELF2	0.630208175	4.96E-231	SMC2
TNFRSF12A	0.628389521	1.59E-225	SMC2
LTBP4	0.54621265	4.02E-225	SMC2
IL17B	0.826028908	7.24E-225	SMC2
PRSS23	0.640040781	5.82E-224	SMC2
MATN2	0.528653016	2.18E-220	SMC2
MYBL1	0.781869774	1.51E-215	SMC2
LPP	0.616231471	4.05E-204	SMC2
EMB	0.618757662	1.60E-202	SMC2
GNG11	0.754783427	3.22E-196	SMC2
MTUS2	0.518155541	1.48E-195	SMC2
MFAP2	0.668708048	1.14E-170	SMC2
SYNPO2	0.524488555	8.86E-168	SMC2
LTBP1	0.560457273	4.69E-163	SMC2
TCAP	0.505564731	1.76E-162	SMC2
COPZ2	0.513062441	1.34E-160	SMC2
CNN1	0.638984587	4.66E-158	SMC2
PNCK	0.564595378	1.53E-157	SMC2
WWP2	0.620403357	8.13E-153	SMC2
ARHGAP5	0.568677228	6.84E-148	SMC2
FN1	0.627547527	5.97E-138	SMC2
ERRFI1	0.70108162	6.43E-134	SMC2
WWTR1	0.525537935	3.66E-126	SMC2
FHL1	0.50458774	1.50E-117	SMC2
TNC	1.404812839	0	SMC3
GSN	0.528705386	5.29E-265	SMC3
CCN3	0.715927205	2.37E-238	SMC3
ITIH4	0.725033271	2.61E-220	SMC3

© 2022. Shi X et al. Published online at http://www.aginganddisease.org/EN/10.14336/AD.2022.0313

PRSS23	0.605372843	2.20E-214	SMC3
ENSCAFG00000018277	0.567737436	1.44E-123	SMC3
RGS2	0.581219562	2.27E-122	SMC3
APOA1	2.752136287	0	SMC4
THY1	1.947985921	0	SMC4
REV3L	1.916930393	0	SMC4
TNXB	1.391727649	0	SMC4
HMCN2	1.304840726	0	SMC4
HPD	0.826605941	5.82E-296	SMC4
ENSCAFG00000000701	1.941210809	1.14E-290	SMC4
CCL7	2.271206379	2.05E-289	SMC4
ICAM1	1.562037508	3.56E-275	SMC4
GUCY1A2	1.474429208	6.23E-274	SMC4
ADAMTS4	1.584272556	1.34E-272	SMC4
COL14A1	1.496893314	1.75E-255	SMC4
PRSS35	0.77912057	2.86E-253	SMC4
EGFLAM	0.612870008	6.11E-249	SMC4
DAAM1	1.274742618	1.52E-248	SMC4
ADRA2A	2.030603882	6.75E-244	SMC4
ENSCAFG00000031706	1.517911086	5.27E-232	SMC4
IGFBP5	1.706226588	4.65E-222	SMC4
SASH1	1.025301204	1.74E-219	SMC4
ECRG4	0.718119798	5.60E-218	SMC4
FST	1.170832815	2.84E-215	SMC4
FABP4	1.700665153	1.50E-209	SMC4
PLAU	1.408482551	4.81E-199	SMC4
ZEB2	1.161510729	4.95E-193	SMC4
ASPN	0.879259047	1.13E-190	SMC4
PMP22	1.116967669	8.11E-190	SMC4
RGS16	1.532257516	1.55E-180	SMC4
REL	0.568229711	3.71E-179	SMC4
MYH9	0.934323856	5.47E-175	SMC4
TPPP3	0.916353619	6.42E-173	SMC4
CLEC3B	1.546953892	9.34E-171	SMC4
PDE4B	0.988064476	3.93E-166	SMC4
NR2F2	1.033018599	1.67E-165	SMC4
B2M	0.77499332	4.34E-156	SMC4
GLUL	1.025778746	3.52E-141	SMC4
IL33	1.232444245	8.35E-138	SMC4
CD36	0.962615761	1.31E-136	SMC4
COL6A3	1.072558046	1.86E-128	SMC4
ENSCAFG00000023111	0.974713663	4.93E-128	SMC4
TMEM176A	0.927318712	3.25E-126	SMC4
LTBP4	0.915991886	4.12E-123	SMC4
DLA88	0.655270118	2.17E-121	SMC4

PRRX1	0.785395687	9.18E-116	SMC4
PCOLCE	0.911979454	3.08E-113	SMC4
VEGFA	0.606290811	3.29E-105	SMC4
CP	0.950370653	3.97E-100	SMC4
LAMC3	0.70145855	2.86E-98	SMC4
LGALS3	0.699743551	1.21E-92	SMC4
AQP1	0.654771295	8.05E-91	SMC4
REM1	0.774318701	2.12E-89	SMC4
COL1A1	0.880355027	1.56E-85	SMC4
RARRES2	0.526222106	1.30E-84	SMC4
NDUFA4L2	0.812191992	2.06E-80	SMC4
MGP	0.660189314	2.64E-79	SMC4
FRMD3	0.571772238	1.15E-78	SMC4
GNG11	0.661165804	8.17E-73	SMC4
C1orf54	0.600777316	2.46E-64	SMC4
DDIT4	0.697262549	3.74E-57	SMC4
ANXA2	0.508711738	1.15E-55	SMC4
ADGRF5	0.767651807	1.38E-55	SMC4
ADAMTS1	0.514108556	9.43E-55	SMC4
HIGD1B	0.62755876	6.74E-52	SMC4
S100A5	0.559883231	8.17E-52	SMC4
RGS5	0.61543544	8.72E-52	SMC4
FXYD6	0.657540728	1.55E-48	SMC4
IL1R1	0.509423591	2.11E-48	SMC4
GADD45A	0.559861358	2.35E-47	SMC4
CXCL12	0.605607543	7.89E-47	SMC4
H2AZ1	0.520330063	4.46E-46	SMC4
ENSCAFG00000025063	0.544570091	7.49E-44	SMC4
C7	0.709457527	4.79E-42	SMC4
NFKBIA	0.970429567	1.00E-41	SMC4
PI3	1.079356344	6.81E-41	SMC4
CLU	0.568769303	8.34E-41	SMC4
C1R	0.783668669	6.82E-39	SMC4
FGF7	0.722700969	7.34E-39	SMC4
TIMP3	0.557967538	1.12E-37	SMC4
MDFIC	0.538444974	3.34E-36	SMC4
C1S	0.802001263	8.52E-36	SMC4
VTN	0.605465612	8.58E-32	SMC4
SPTBN1	0.599894571	4.64E-30	SMC4
EDNRB	0.526207179	6.99E-30	SMC4
DNAJB1	0.631589033	1.22E-27	SMC4
EPHA2	0.555965595	2.44E-26	SMC4
RALGPS2	0.761851173	5.03E-26	SMC4
RHOJ	0.643084397	1.75E-22	SMC4
ARHGAP45	1.236727942	8.23E-21	SMC4

© 2022. Shi X et al. Published online at http://www.aginganddisease.org/EN/10.14336/AD.2022.0313

VCAM1	0.56257903	3.96E-18	SMC4
UGCG	0.540331404	5.06E-17	SMC4
HGF	0.777136061	8.71E-17	SMC4
COL3A1	0.576766517	9.58E-17	SMC4
DCN	1.207481268	1.35E-16	SMC4
COL12A1	0.582059048	9.48E-16	SMC4
RASD1	0.567484847	1.03E-10	SMC4
ENSCAFG00000010290	0.712053853	3.54E-08	SMC4
PDGFRL	0.602567681	1.27E-05	SMC4
APOLD1	0.719604605	0.000290289	SMC4

Supplementary Table 6. List of highly variable genes for the two MFB subsets in beagle dogs.

Gene	Log_2(fold change)	Adjusted *P*	Cluster
LTBP1	1.006002	4.28E-76	MFB1
VCAN	1.151206	4.23E-63	MFB1
POSTN	1.817134	1.65E-61	MFB1
PRG4	1.762531	1.25E-50	MFB1
CD109	0.889934	2.30E-49	MFB1
S100A5	1.161506	1.14E-47	MFB1
IGFBP7	0.678684	8.08E-47	MFB1
COL14A1	1.01136	5.36E-46	MFB1
CAPG	0.936727	1.86E-44	MFB1
AQP1	1.020885	1.08E-43	MFB1
FAP	0.708934	2.00E-43	MFB1
BGN	0.625854	3.35E-41	MFB1
RELN	0.533167	2.70E-36	MFB1
FXYD5	0.681177	7.43E-35	MFB1
APOE	0.710557	4.23E-33	MFB1
PCSK1N	0.679623	2.11E-32	MFB1
TIMP1	0.974924	2.58E-32	MFB1
NDUFA4L2	1.01562	8.90E-32	MFB1
PMEPA1	0.631871	7.95E-30	MFB1
MGP	0.572226	1.45E-29	MFB1
FN1	0.709722	1.06E-27	MFB1
UACA	0.606601	5.27E-26	MFB1
IFI6	0.746862	6.04E-24	MFB1
DPT	0.715597	2.90E-22	MFB1
PAM	0.512819	6.38E-22	MFB1
COL5A2	0.530317	2.50E-17	MFB1
COMP	1.006808	1.41E-11	MFB1
CLU	0.6957	1.78E-09	MFB1
CCN3	0.839369	5.70E-09	MFB1
CCL7	0.502496	0.016652779	MFB1

© 2022. Shi X et al. Published online at http://www.aginganddisease.org/EN/10.14336/AD.2022.0313

MYL9	2.056391	5.33E-132	MFB2
ACTA2	1.78172	4.91E-119	MFB2
ENSCAFG00000018277	1.772401	8.59E-119	MFB2
ARHGAP45	3.161425	1.09E-111	MFB2
ENSCAFG00000010290	1.342177	1.73E-109	MFB2
DCN	1.864973	3.75E-108	MFB2
TPM2	1.518811	4.06E-104	MFB2
DGUOK	1.989804	6.44E-100	MFB2
DSTN	1.204441	4.72E-98	MFB2
MFAP5	1.223213	3.99E-91	MFB2
PLN	1.170443	3.38E-88	MFB2
PPP1R14A	1.502781	4.23E-85	MFB2
RCAN2	1.003655	1.05E-84	MFB2
MYH11	1.407008	2.29E-84	MFB2
ABCA5	1.30407	3.35E-81	MFB2
ENSCAFG00000042554	0.622642	1.71E-78	MFB2
PPP1R12A	0.911844	5.26E-77	MFB2
ACTN4	0.943861	4.47E-75	MFB2
CXCL12	1.195032	2.96E-74	MFB2
RAMP1	1.149652	3.33E-74	MFB2
TPM1	1.075398	5.33E-73	MFB2
CLEC3B	0.915623	1.82E-71	MFB2
GADD45G	1.641766	6.50E-70	MFB2
BCAM	1.109858	1.35E-68	MFB2
SERPINF1	0.856533	1.56E-68	MFB2
C1S	0.909888	1.23E-61	MFB2
TINAGL1	0.880282	4.36E-61	MFB2
PIF1	0.910701	8.59E-59	MFB2
CYCS	1.176115	4.86E-58	MFB2
ENSCAFG00000028930	2.330941	1.72E-57	MFB2
NEXN	0.951447	3.11E-57	MFB2
SMTN	0.971801	3.93E-57	MFB2
GSN	0.620909	1.50E-56	MFB2
HSPB1	0.675468	7.42E-55	MFB2
WFDC1	0.758165	1.31E-54	MFB2
PI3	1.688718	1.73E-54	MFB2
SORBS2	0.818611	4.85E-53	MFB2
SVIL	1.060662	6.76E-53	MFB2
C1R	0.692621	7.40E-50	MFB2
ITGA7	0.64555	1.15E-49	MFB2
AKAP12	0.912804	1.22E-49	MFB2
NTRK2	0.811754	6.55E-49	MFB2
ITIH5	0.545517	5.83E-47	MFB2
ITGBL1	0.712719	1.46E-46	MFB2
GADD45B	1.393942	6.11E-46	MFB2

© 2022. Shi X et al. Published online at http://www.aginganddisease.org/EN/10.14336/AD.2022.0313

FILIP1L	0.893818	7.28E-46	MFB2
RARRES2	0.59577	7.82E-46	MFB2
COX6A2	0.756814	8.02E-46	MFB2
ROCK1	0.634042	1.65E-44	MFB2
SLC25A4	0.668072	1.50E-42	MFB2
PNCK	0.757318	2.55E-42	MFB2
UBA2	0.741394	8.88E-41	MFB2
MYLK	0.67788	1.78E-39	MFB2
MT1E	2.768207	1.33E-38	MFB2
FOS	1.090975	1.41E-38	MFB2
PLAU	0.943101	1.97E-38	MFB2
NR4A1	1.321037	2.09E-38	MFB2
PCOLCE2	0.822944	2.93E-38	MFB2
CELF2	0.577869	3.73E-38	MFB2
PDE6H	0.548149	1.05E-37	MFB2
PTP4A3	1.229489	4.91E-37	MFB2
FAM13C	0.554858	3.93E-36	MFB2
TESC	1.04718	2.93E-35	MFB2
RGS16	0.718125	7.79E-35	MFB2
RBP4	0.564582	1.23E-34	MFB2
RBPMS	0.520918	2.80E-34	MFB2
SLMAP	0.6108	3.39E-34	MFB2
EGR1	0.821542	4.76E-34	MFB2
ANGPTL1	0.594103	1.32E-33	MFB2
BTG2	0.990721	2.24E-33	MFB2
TLN1	0.562313	2.55E-33	MFB2
SFRP4	1.268275	1.41E-32	MFB2
MSRB3	0.538568	2.55E-32	MFB2
TAGLN	0.544073	2.79E-32	MFB2
ECM1	0.566313	3.18E-32	MFB2
ARHGAP29	0.654666	6.14E-32	MFB2
FOSB	0.78792	7.86E-32	MFB2
ZFP36	0.642014	1.48E-31	MFB2
SVEP1	0.689016	6.92E-31	MFB2
ATF3	1.18151	2.00E-30	MFB2
PDGFRL	0.539428	2.43E-30	MFB2
CHRDL1	0.59565	3.16E-30	MFB2
MSRB1	0.642165	4.11E-30	MFB2
ENSCAFG00000025332	0.612456	2.96E-29	MFB2
RRAD	0.517971	1.07E-28	MFB2
CD248	0.724774	1.62E-28	MFB2
ENSCAFG00000023594	0.523541	2.93E-28	MFB2
RHOB	0.537989	1.53E-27	MFB2
CNN1	0.580447	2.15E-26	MFB2
PPP1R15A	0.670937	1.35E-25	MFB2

© 2022. Shi X et al. Published online at http://www.aginganddisease.org/EN/10.14336/AD.2022.0313

SYNE2	0.823873	1.08E-24	MFB2
RASL11A	0.570814	1.15E-24	MFB2
IRF1	0.543237	1.46E-23	MFB2
ABI3BP	0.532895	3.13E-23	MFB2
DMPK	0.509439	1.16E-21	MFB2
IGFBP5	0.610182	1.22E-21	MFB2
GADD45A	0.710315	1.31E-19	MFB2
SLC22A3	0.638332	1.89E-19	MFB2
SLC30A1	0.906545	8.49E-19	MFB2
COX4I2	0.943452	1.20E-18	MFB2
CKM	0.505072	7.39E-18	MFB2
MGLL	0.669398	1.24E-16	MFB2
FGL1	0.855286	3.63E-14	MFB2
ETS2	0.513372	6.91E-14	MFB2
MT2A	1.974998	1.70E-11	MFB2
CPZ	0.650344	3.21E-11	MFB2
CACNA1H	0.503228	5.21E-10	MFB2
MYOC	0.952119	1.74E-08	MFB2
COL1A1	0.73117	3.09E-08	MFB2
LAMA2	0.550064	2.79E-07	MFB2
IL6	0.644032	0.000849432	MFB2

Supplementary Table 7. Up-regulated genes in TNC$^+$ SMC subset of AS-prone artery from 3S-ASCVD dog.

Gene	Log_2(fold change)	Adjusted *P*
MGP	0.85255	5.10E-60
POSTN	0.96848	7.46E-45
VCAN	0.63192	2.69E-41
COL14A1	0.76199	1.72E-34
SMOC2	0.76562	1.15E-32
CLU	0.80307	2.51E-32
SAA1	0.74178	4.28E-30
LGALS3	0.5052	1.87E-26
ENSCAFG00000015206	0.58996	7.86E-26
FBN1	0.5549	2.66E-24
NDUFA4L2	0.69143	1.61E-08

Supplementary Table 8. List of highly variable genes for the non-immune cell subsets in human carotid plaques.

**Gene**	**log_2(fold change)**	**Adjusted *P***	**Cluster**
**SMCs**			
FRZB	1.313	1.92E-120	SMC1
RAMP1	0.988	3.22E-112	SMC1

© 2022. Shi X et al. Published online at http://www.aginganddisease.org/EN/10.14336/AD.2022.0313

CLU	1.231	3.95E-108	SMC1
SCRG1	1.023	1.35E-82	SMC1
ITGA8	0.672	2.16E-79	SMC1
TCEAL2	0.675	4.23E-74	SMC1
PLN	0.919	5.61E-71	SMC1
SOD3	0.593	3.91E-69	SMC1
MFAP4	0.693	1.67E-58	SMC1
FXYD1	0.542	2.64E-57	SMC1
DSTN	0.553	1.38E-56	SMC1
C12orf75	0.684	3.75E-55	SMC1
SPINT2	0.569	1.90E-52	SMC1
CPE	0.543	2.89E-52	SMC1
NFIA	0.532	3.46E-50	SMC1
SOST	0.909	7.39E-49	SMC1
MYH11	0.600	1.50E-48	SMC1
PRDM6	0.525	4.94E-48	SMC1
PCDH7	0.512	1.16E-44	SMC1
PDE5A	0.745	2.81E-43	SMC1
LMCD1	0.596	9.48E-43	SMC1
MT1X	0.802	8.89E-40	SMC1
CCDC3	0.564	8.82E-39	SMC1
SORBS1	0.576	3.47E-38	SMC1
GADD45G	0.725	1.62E-35	SMC1
ACTC1	0.704	9.57E-35	SMC1
NRGN	0.618	7.36E-32	SMC1
THSD4	0.597	8.26E-32	SMC1
NET1	0.518	2.66E-31	SMC1
CSRP2	0.534	8.96E-30	SMC1
RRAD	0.551	4.47E-29	SMC1
DLX6-AS1	0.615	2.60E-28	SMC1
JUNB	0.593	6.36E-21	SMC1
IER2	0.587	7.51E-21	SMC1
C11orf96	0.612	4.29E-17	SMC1
FOS	0.546	6.72E-17	SMC1
JUN	0.529	4.20E-16	SMC1
ZFP36	0.524	1.34E-14	SMC1
SUCNR1	0.703	6.10E-13	SMC1
SOCS3	0.583	1.14E-11	SMC1
GADD45B	0.560	4.68E-08	SMC1
IRF1	0.523	3.04E-02	SMC1
FN1	1.270	8.53E-141	SMC2
CFH	1.262	1.02E-126	SMC2
PRSS23	1.196	1.32E-120	SMC2
VCAN	1.037	6.83E-119	SMC2
SULF1	1.033	4.08E-118	SMC2
COL15A1	1.119	1.59E-113	SMC2

© 2022. Shi X et al. Published online at http://www.aginganddisease.org/EN/10.14336/AD.2022.0313

CCDC80	1.574	1.62E-110	SMC2
LUM	2.133	3.07E-109	SMC2
COL1A2	1.486	7.10E-106	SMC2
FBLN2	0.982	6.70E-105	SMC2
AEBP1	0.802	4.46E-104	SMC2
F2R	0.964	2.60E-102	SMC2
TMSB10	0.699	5.37E-101	SMC2
POSTN	1.980	7.27E-92	SMC2
MMP23B	0.723	1.29E-91	SMC2
THBS2	0.911	4.89E-86	SMC2
SFRP2	2.077	8.44E-85	SMC2
TIMP1	1.163	5.54E-80	SMC2
COL3A1	1.724	4.37E-76	SMC2
COL1A1	1.849	4.49E-74	SMC2
SERPINE1	1.065	2.27E-73	SMC2
PLAC9	0.710	6.11E-73	SMC2
PCOLCE2	0.769	2.57E-72	SMC2
SHISA3	0.594	8.80E-71	SMC2
EFEMP1	0.828	1.50E-70	SMC2
LTBP2	0.791	3.85E-66	SMC2
SPARC	0.888	3.17E-64	SMC2
MGP	0.670	6.10E-63	SMC2
KRT17	0.940	3.41E-62	SMC2
COL6A2	0.577	7.58E-62	SMC2
INHBA	0.753	7.53E-61	SMC2
SERPINE2	1.232	2.12E-59	SMC2
BGN	0.541	1.23E-55	SMC2
MMP2	0.716	5.16E-55	SMC2
KRT7	0.705	9.28E-55	SMC2
GAP43	0.635	1.76E-54	SMC2
COL4A1	0.513	6.25E-54	SMC2
CTSZ	0.536	2.80E-53	SMC2
SUGCT	0.636	7.45E-53	SMC2
CXCL12	0.731	8.15E-53	SMC2
COL4A2	0.535	9.14E-53	SMC2
COL5A1	0.762	9.24E-52	SMC2
ISLR	0.649	5.02E-51	SMC2
CDH11	0.656	3.49E-49	SMC2
CRLF1	0.689	1.04E-48	SMC2
TNFRSF11B	0.803	1.14E-48	SMC2
PLPP1	0.601	1.82E-48	SMC2
FSTL1	0.613	4.63E-48	SMC2
FAP	0.622	1.03E-47	SMC2
ANTXR1	0.623	1.32E-47	SMC2
C1R	0.567	5.34E-46	SMC2
PCOLCE	0.657	1.25E-45	SMC2

© 2022. Shi X et al. Published online at http://www.aginganddisease.org/EN/10.14336/AD.2022.0313

MARCKS	0.594	2.54E-45	SMC2
FBN1	0.529	2.39E-41	SMC2
THBS1	0.939	2.54E-41	SMC2
C5orf46	0.595	3.47E-38	SMC2
SFRP4	0.755	4.81E-38	SMC2
MRC2	0.526	6.81E-38	SMC2
HTRA1	0.613	8.06E-38	SMC2
COL5A2	0.697	3.11E-37	SMC2
CRTAC1	0.614	3.24E-37	SMC2
ENG	0.595	1.61E-36	SMC2
KRT18	0.655	2.12E-36	SMC2
COL8A1	0.607	8.76E-34	SMC2
GGT5	0.561	9.79E-34	SMC2
PPIC	0.509	2.49E-33	SMC2
IL32	0.511	8.50E-33	SMC2
COL6A3	0.693	7.09E-32	SMC2
ADH1B	0.653	1.60E-31	SMC2
NDUFA4L2	0.578	3.00E-31	SMC2
KRT14	0.775	3.87E-31	SMC2
AKAP12	0.571	4.59E-31	SMC2
PCDH10	0.576	8.62E-30	SMC2
IGFBP3	0.749	1.96E-29	SMC2
MFAP5	0.856	2.48E-29	SMC2
FBLN1	0.699	7.28E-28	SMC2
COL12A1	0.512	1.11E-27	SMC2
DCN	1.015	6.53E-27	SMC2
CTHRC1	0.750	5.70E-24	SMC2
ANKRD1	0.533	1.34E-18	SMC2
S100A10	0.520	1.80E-18	SMC2
SRGN	0.545	2.60E-18	SMC2
GAS6	0.802	6.44E-17	SMC2
ELN	0.731	3.66E-16	SMC2
PTGDS	0.857	1.63E-12	SMC2
IFI6	0.534	2.07E-08	SMC2
COX4I2	1.044	2.92E-176	SMC3
PLXDC1	1.139	3.41E-157	SMC3
CPM	1.179	1.74E-156	SMC3
OLFML2A	1.032	4.62E-133	SMC3
NEURL1B	0.710	4.82E-115	SMC3
OR51E1	0.699	1.65E-111	SMC3
CCDC102B	1.291	3.50E-108	SMC3
APOLD1	0.914	9.53E-103	SMC3
ENPEP	0.620	6.09E-93	SMC3
CDH6	0.830	8.57E-91	SMC3
ADGRF5	1.087	4.53E-87	SMC3
PLPPR4	0.558	2.86E-82	SMC3

© 2022. Shi X et al. Published online at http://www.aginganddisease.org/EN/10.14336/AD.2022.0313

SEMA5B	0.566	2.72E-75	SMC3
AVPR1A	0.535	5.30E-75	SMC3
ADAMTS4	0.985	7.84E-74	SMC3
KLHL23	0.740	2.81E-71	SMC3
CRISPLD2	0.948	2.16E-68	SMC3
HIGD1B	0.528	4.62E-67	SMC3
LZTS1	0.600	1.25E-66	SMC3
GUCY1A2	0.787	1.20E-62	SMC3
SCIN	0.547	4.98E-59	SMC3
ABCC9	1.061	1.39E-50	SMC3
GPR4	0.567	2.32E-50	SMC3
CD36	0.789	1.76E-49	SMC3
TPPP3	0.833	9.77E-49	SMC3
HLA-B	1.067	1.47E-48	SMC3
NOTCH3	1.332	1.51E-46	SMC3
CHN1	0.800	3.56E-45	SMC3
B2M	0.826	4.75E-45	SMC3
SERPINF1	0.898	5.66E-44	SMC3
COL18A1	1.257	5.81E-44	SMC3
KCNJ8	0.626	1.36E-43	SMC3
IGFBP4	1.086	1.56E-43	SMC3
ADAMTS9	0.527	3.52E-42	SMC3
MCAM	1.184	6.86E-42	SMC3
COL5A3	0.564	1.07E-40	SMC3
ADCY3	0.581	1.79E-40	SMC3
CD248	0.821	5.34E-40	SMC3
SPON2	0.758	2.56E-39	SMC3
GJA4	0.863	2.11E-37	SMC3
GJC1	0.662	3.39E-37	SMC3
THY1	1.853	4.63E-37	SMC3
COL4A1	1.325	1.51E-36	SMC3
MT-CO2	0.826	2.59E-35	SMC3
GUCY1B1	0.809	3.81E-35	SMC3
TGFBI	0.971	7.43E-35	SMC3
IFI27	1.212	6.14E-34	SMC3
OLFML2B	0.928	1.39E-33	SMC3
TBX2	0.889	1.73E-33	SMC3
PDGFRB	1.035	3.68E-33	SMC3
PHLDA1	1.125	4.68E-32	SMC3
SLC7A2	0.756	1.61E-31	SMC3
SFTA1P	0.694	2.80E-31	SMC3
COL4A2	1.003	3.17E-31	SMC3
HLA-A	0.676	1.07E-30	SMC3
HES4	0.781	1.39E-29	SMC3
NID1	0.672	7.66E-29	SMC3
MALAT1	0.661	1.03E-28	SMC3

© 2022. Shi X et al. Published online at http://www.aginganddisease.org/EN/10.14336/AD.2022.0313

SUPPLEMENTARY DATA

CYTOR	1.159	1.79E-28	SMC3
TINAGL1	0.775	3.56E-27	SMC3
LOXL2	0.640	1.68E-26	SMC3
IFITM3	0.628	3.42E-26	SMC3
STEAP4	0.937	5.28E-26	SMC3
EPAS1	0.833	2.27E-25	SMC3
IFITM1	1.041	6.84E-25	SMC3
MARCKSL1	0.548	1.98E-24	SMC3
IFITM2	0.620	2.20E-24	SMC3
AGRN	0.649	3.10E-24	SMC3
ADGRA2	0.701	3.45E-24	SMC3
ITGA1	0.819	8.34E-24	SMC3
EBF1	0.770	9.04E-24	SMC3
FABP5	0.703	9.50E-24	SMC3
RASD1	0.685	9.70E-24	SMC3
MAF	0.660	1.10E-23	SMC3
ANGPT2	0.618	1.35E-22	SMC3
EHD2	0.676	2.10E-22	SMC3
ZEB2	0.630	4.35E-22	SMC3
NDUFA4L2	0.693	8.64E-22	SMC3
KCNE4	0.657	9.08E-22	SMC3
ENTPD1	0.577	1.44E-21	SMC3
INPP4B	0.809	2.09E-21	SMC3
PGF	0.767	3.00E-21	SMC3
TFPI	0.900	3.42E-21	SMC3
EDNRA	0.564	4.86E-21	SMC3
TIMP3	0.924	5.00E-21	SMC3
MIR4435-2HG	0.860	6.72E-21	SMC3
MT2A	1.107	7.28E-21	SMC3
ETS1	0.629	1.62E-20	SMC3
ASAP1	0.571	1.79E-20	SMC3
SLC12A2	0.609	5.97E-20	SMC3
MT-CO1	0.599	2.48E-19	SMC3
LAMA4	0.682	4.76E-19	SMC3
ITGA7	0.604	1.11E-18	SMC3
SLIT3	0.722	1.74E-18	SMC3
LAMA5	0.668	2.47E-18	SMC3
HLA-C	0.584	3.80E-18	SMC3
MT-CO3	0.585	4.31E-17	SMC3
OAZ2	0.585	3.22E-16	SMC3
MYO1B	0.661	4.38E-16	SMC3
PTP4A3	0.630	9.01E-16	SMC3
AGT	0.645	9.79E-16	SMC3
PDLIM1	0.611	1.69E-15	SMC3
HIF1A	0.644	8.21E-15	SMC3
LAMC1	0.647	1.82E-14	SMC3

© 2022. Shi X et al. Published online at http://www.aginganddisease.org/EN/10.14336/AD.2022.0313

SLCO3A1	0.614	2.37E-14	SMC3
UACA	0.669	3.28E-14	SMC3
4-Sep	0.657	5.25E-14	SMC3
PHLDA2	0.949	1.23E-13	SMC3
ISG15	0.766	1.33E-13	SMC3
KRT18	0.505	1.71E-13	SMC3
EPS8	0.560	1.94E-13	SMC3
UGCG	0.579	2.02E-13	SMC3
ZFP36L1	0.640	2.95E-13	SMC3
NES	0.536	4.16E-13	SMC3
VMP1	0.673	7.10E-13	SMC3
SPARCL1	0.799	1.64E-12	SMC3
FAM13C	0.503	3.51E-12	SMC3
STOM	0.549	4.03E-12	SMC3
LHFPL6	0.508	6.29E-12	SMC3
IFI16	0.553	9.13E-12	SMC3
APOE	0.689	3.18E-11	SMC3
IFI6	0.658	6.36E-11	SMC3
RGS5	0.588	7.97E-11	SMC3
TNC	0.540	8.58E-11	SMC3
SDC2	0.585	1.10E-10	SMC3
JAG1	0.643	1.40E-10	SMC3
SYTL2	0.500	1.62E-10	SMC3
ANKRD28	0.524	6.09E-10	SMC3
FAT1	0.659	6.42E-10	SMC3
CCND1	0.808	3.52E-09	SMC3
ISYNA1	0.522	8.00E-08	SMC3
ARHGAP1	0.509	1.33E-07	SMC3
PDGFA	0.582	5.09E-07	SMC3
NRP1	0.522	8.05E-07	SMC3
KLF9	0.517	1.04E-06	SMC3
SERPINH1	0.615	1.77E-06	SMC3
LASP1	0.591	6.11E-06	SMC3
MT1E	0.670	6.94E-05	SMC3
MYLK	0.640	3.44E-04	SMC3
BCAM	0.523	4.15E-04	SMC3
LBH	0.622	4.27E-03	SMC3
**ECs**			
COL15A1	1.932	2.29E-23	EC1
SPRY1	1.475	5.60E-18	EC1
A2M	1.079	5.99E-15	EC1
SPARCL1	1.141	4.49E-14	EC1
GNG11	1.111	1.54E-13	EC1
COL4A2	1.256	1.48E-12	EC1
TSHZ2	0.878	5.29E-12	EC1

© 2022. Shi X et al. Published online at http://www.aginganddisease.org/EN/10.14336/AD.2022.0313

APOLD1	1.187	5.73E-12	EC1
HLA-B	0.594	9.74E-12	EC1
COL4A1	1.351	4.34E-11	EC1
APLNR	1.029	3.76E-10	EC1
S100A10	0.608	4.51E-10	EC1
EMCN	0.935	4.85E-10	EC1
CLEC2B	0.951	5.18E-10	EC1
ITGA6	0.672	7.30E-10	EC1
ZNF385D	0.827	1.73E-09	EC1
COL6A2	0.975	2.97E-09	EC1
RGCC	1.314	3.78E-09	EC1
ACKR1	1.431	5.41E-09	EC1
PMP22	0.936	6.21E-09	EC1
SELE	1.883	1.08E-08	EC1
FABP5	1.490	1.40E-08	EC1
HLA-A	0.594	3.59E-08	EC1
CXorf36	0.748	4.42E-08	EC1
NFKBIA	0.949	1.94E-07	EC1
COTL1	0.913	2.03E-07	EC1
CST3	0.766	2.80E-07	EC1
KLF6	0.738	3.10E-07	EC1
MARCKS	0.853	2.57E-06	EC1
CD93	0.779	3.71E-06	EC1
LINC01235	0.617	1.23E-05	EC1
ANKRD28	0.828	4.72E-05	EC1
ETS1	0.742	4.75E-05	EC1
HSPA1A	0.601	5.59E-05	EC1
RASAL2	0.543	5.81E-05	EC1
ADGRL4	0.671	6.44E-05	EC1
FAM167B	0.833	7.09E-05	EC1
GMFG	0.700	8.54E-05	EC1
IFI27	0.583	1.61E-04	EC1
TM4SF18	0.702	2.18E-04	EC1
ZFP36L1	0.745	2.37E-04	EC1
KDR	0.573	3.08E-04	EC1
DNAJB1	0.644	6.96E-04	EC1
CTHRC1	0.673	7.43E-04	EC1
ARL4C	0.629	1.02E-03	EC1
CD200	0.518	2.33E-03	EC1
DNASE1L3	0.634	3.79E-03	EC1
MEF2C	0.663	3.89E-03	EC1
RGS16	0.750	4.25E-03	EC1
GYPC	0.636	4.60E-03	EC1
ADAMTS9	0.656	6.60E-03	EC1
PCDH17	0.789	6.69E-03	EC1
ENTPD1	0.619	7.85E-03	EC1

© 2022. Shi X et al. Published online at http://www.aginganddisease.org/EN/10.14336/AD.2022.0313

INSR	0.863	8.20E-03	EC1
NUAK1	0.711	1.12E-02	EC1
ICAM1	0.978	1.82E-02	EC1
TNFAIP3	0.889	2.15E-02	EC1
HSPH1	0.675	3.68E-02	EC1
C2CD4B	0.832	3.71E-02	EC1
STC1	1.492	3.96E-02	EC1
OMD	1.934	7.49E-69	EC2
COL8A1	1.180	2.60E-60	EC2
GDF7	1.341	9.36E-60	EC2
PTGIS	1.172	3.20E-58	EC2
GJA5	1.217	3.39E-58	EC2
HHIP	1.331	1.23E-57	EC2
SULF1	1.719	1.03E-56	EC2
OGN	2.136	1.50E-55	EC2
LTBP4	1.253	1.16E-53	EC2
BMP6	0.855	4.21E-51	EC2
NRG1	1.033	1.13E-49	EC2
DKK2	0.964	1.56E-48	EC2
BMX	1.173	2.02E-47	EC2
PROCR	1.385	1.09E-45	EC2
NDRG4	0.635	4.62E-43	EC2
ITLN1	2.266	2.00E-42	EC2
PLCXD3	0.797	4.58E-42	EC2
ELN	2.003	5.45E-42	EC2
BMP4	1.253	5.08E-40	EC2
F5	0.633	3.75E-39	EC2
SRPX	0.608	1.59E-38	EC2
PCDH7	0.681	5.07E-38	EC2
CPE	1.335	2.30E-37	EC2
FBLIM1	0.903	4.30E-37	EC2
PLXNA4	0.583	7.47E-37	EC2
CP	1.170	8.08E-37	EC2
C1S	1.040	1.05E-36	EC2
HMCN1	1.057	1.53E-36	EC2
LEPR	0.987	2.77E-36	EC2
SLPI	1.346	6.80E-36	EC2
EFEMP1	1.787	3.75E-35	EC2
TMEM120A	0.759	4.95E-35	EC2
CDH11	0.903	1.12E-34	EC2
EDN1	1.891	2.16E-34	EC2
POU4F1	0.594	4.58E-34	EC2
SPINT2	0.685	1.86E-33	EC2
MRC2	0.537	3.28E-33	EC2
C1R	0.975	5.85E-31	EC2
BGN	1.330	1.97E-29	EC2

© 2022. Shi X et al. Published online at http://www.aginganddisease.org/EN/10.14336/AD.2022.0313

ANXA3	0.587	4.42E-29	EC2
MEIS2	0.732	1.84E-28	EC2
CLU	1.388	1.69E-27	EC2
IL1RL1	0.973	2.47E-27	EC2
DHRS3	0.827	7.02E-27	EC2
GMDS	0.577	8.18E-27	EC2
PAPSS2	0.618	9.23E-27	EC2
CELF2	0.615	2.54E-26	EC2
CYP1A1	0.887	2.71E-26	EC2
SELP	0.960	4.12E-26	EC2
FKBP5	0.781	9.32E-26	EC2
RUNX1T1	0.705	1.35E-25	EC2
FN1	1.273	1.50E-25	EC2
BMPER	0.617	2.54E-25	EC2
FGF18	0.503	4.65E-25	EC2
C19orf33	0.725	6.01E-25	EC2
LTBP2	0.881	1.33E-24	EC2
NUPR1	0.899	1.39E-24	EC2
SELL	0.947	1.76E-24	EC2
CRTAC1	1.187	3.05E-24	EC2
PTGS2	1.244	4.78E-24	EC2
RBMS3	0.859	1.12E-23	EC2
WSB1	0.899	1.32E-23	EC2
CRIM1	1.125	1.39E-23	EC2
MPZL2	0.772	9.07E-23	EC2
MIR99AHG	0.567	3.84E-22	EC2
FAM107B	0.640	4.61E-22	EC2
TBX1	0.589	6.20E-22	EC2
MGP	1.342	1.26E-21	EC2
ITPR2	0.536	1.67E-21	EC2
TXNDC5	0.581	4.25E-21	EC2
GAS6	0.852	4.56E-20	EC2
LTBP3	0.648	1.16E-19	EC2
LMO2	0.741	3.34E-19	EC2
HRCT1	0.539	5.98E-19	EC2
CTNNAL1	0.656	7.36E-19	EC2
ALDH1A3	0.653	2.68E-18	EC2
C1QTNF1	0.549	3.00E-18	EC2
AAK1	0.679	3.10E-18	EC2
FOXC1	0.687	3.58E-18	EC2
IGFBP3	1.255	5.93E-18	EC2
TPM2	0.649	1.48E-17	EC2
METRNL	0.509	2.07E-17	EC2
PTPRJ	0.503	2.29E-17	EC2
TIMP2	0.603	5.06E-17	EC2
PAWR	0.516	1.10E-16	EC2

KCTD12	0.761	1.40E-16	EC2
ABI3BP	0.653	2.25E-16	EC2
PXDC1	0.539	3.11E-16	EC2
ITGA10	0.695	4.45E-16	EC2
VWF	0.815	6.36E-16	EC2
LTC4S	0.752	7.73E-16	EC2
DAPL1	0.608	4.80E-15	EC2
USP53	0.518	8.24E-14	EC2
PLXNA2	0.560	9.39E-14	EC2
ASS1	0.575	1.19E-13	EC2
ID2	0.611	2.62E-13	EC2
IL33	0.594	2.71E-13	EC2
PTPRB	0.636	5.63E-13	EC2
INMT	0.617	2.89E-12	EC2
ADD1	0.544	3.80E-12	EC2
SMAD6	0.564	5.86E-12	EC2
COL3A1	0.518	7.88E-12	EC2
RARRES2	0.535	1.05E-11	EC2
OAZ1	0.523	4.10E-11	EC2
BCAP29	0.597	4.16E-11	EC2
RHOB	0.574	4.88E-10	EC2
ARL6IP1	0.557	7.01E-10	EC2
SYNPO	0.524	7.11E-10	EC2
CEBPD	0.735	1.54E-09	EC2
IL6ST	0.523	1.77E-09	EC2
HSPG2	0.513	2.91E-09	EC2
MMP2	0.762	8.08E-09	EC2
ID1	0.646	2.51E-08	EC2
DCN	0.775	2.95E-08	EC2
LINC01133	0.520	4.40E-08	EC2
DEPP1	1.175	4.77E-08	EC2
TXNIP	0.597	5.04E-08	EC2
ADIRF	0.583	6.67E-07	EC2
ATP13A3	0.601	1.88E-06	EC2
CTGF	0.743	1.92E-06	EC2
TPM1	0.536	7.00E-06	EC2
TAGLN	0.553	1.21E-03	EC2
CYR61	0.526	2.30E-03	EC2
TFPI	0.523	3.13E-03	EC2
CXCL2	0.741	3.37E-03	EC2
S100A4	0.579	7.33E-03	EC2
MMRN1	0.528	1.94E-02	EC2
**MFBs**			
COL6A3	1.038	1.28E-230	MFB1
MXRA5	0.956	1.01E-204	MFB1

© 2022. Shi X et al. Published online at http://www.aginganddisease.org/EN/10.14336/AD.2022.0313

COL5A2	0.689	2.01E-195	MFB1
SPOCK1	0.721	7.33E-191	MFB1
POSTN	1.029	4.81E-188	MFB1
THY1	0.527	4.95E-180	MFB1
COL5A1	0.617	5.79E-165	MFB1
GALNT1	0.534	7.07E-163	MFB1
COL1A1	0.814	7.21E-162	MFB1
DCN	0.844	8.44E-155	MFB1
MRC2	0.565	9.40E-155	MFB1
TNC	0.911	1.10E-150	MFB1
COL1A2	0.697	4.62E-150	MFB1
PCOLCE	0.525	1.42E-147	MFB1
F2R	0.522	3.88E-146	MFB1
LUM	0.780	1.72E-145	MFB1
TENT5A	0.510	1.53E-143	MFB1
FN1	0.739	1.93E-143	MFB1
ITGBL1	0.532	1.28E-141	MFB1
CTHRC1	0.723	3.29E-139	MFB1
GAP43	0.531	6.15E-136	MFB1
ISLR	0.523	6.52E-135	MFB1
COL3A1	0.670	3.19E-126	MFB1
TIMP1	0.778	9.06E-125	MFB1
IGFBP2	0.708	1.21E-124	MFB1
THBS2	0.558	1.59E-116	MFB1
COL15A1	0.588	2.77E-114	MFB1
NTM	0.514	4.76E-113	MFB1
CACNA2D1	0.662	5.76E-107	MFB1
FNDC1	0.672	8.60E-107	MFB1
VCAM1	0.524	5.96E-103	MFB1
KRT17	0.518	7.39E-101	MFB1
THBS1	0.526	1.03E-88	MFB1
SERPINF1	0.508	1.60E-71	MFB1
TIMP3	1.141	1.13E-68	MFB1
CYP1B1	0.559	4.04E-66	MFB1
CEMIP	0.607	2.43E-65	MFB1
MMP11	0.588	9.48E-49	MFB1
SFRP2	0.537	1.01E-47	MFB1
IBSP	0.763	2.86E-33	MFB1
PTGDS	0.661	1.45E-20	MFB1
PLA2G2A	0.697	7.81E-96	MFB2
FTL	0.653	7.87E-44	MFB2

© 2022. Shi X et al. Published online at http://www.aginganddisease.org/EN/10.14336/AD.2022.0313

FCGR3A	0.519	9.90E-30	MFB2
ITLN1	1.702	1.33E-28	MFB2
HLA-DQB1	0.889	7.77E-27	MFB2
MSR1	0.550	7.06E-26	MFB2
C1QC	0.838	6.75E-24	MFB2
RGS5	0.749	2.83E-23	MFB2
CST1	0.508	7.70E-21	MFB2
KRT8	1.074	3.07E-16	MFB2
GPR183	0.666	5.22E-16	MFB2
PLN	0.509	5.44E-08	MFB2
HLA-DQA1	0.662	5.05E-06	MFB2
LGALS3	0.517	5.70E-04	MFB2
LYZ	0.824	1.44E-02	MFB2
TXN	0.538	2.17E-02	MFB2
JCHAIN	1.684	3.85E-02	MFB2
RAMP2	0.651	4.11E-192	MFB3
EMCN	0.705	1.51E-167	MFB3
MGP	1.314	3.38E-157	MFB3
PLVAP	0.868	9.73E-155	MFB3
FLT1	0.630	3.27E-139	MFB3
PROCR	0.746	7.67E-133	MFB3
AKR1C1	0.925	1.90E-131	MFB3
TM4SF1	0.835	5.89E-120	MFB3
CAVIN2	0.652	4.04E-111	MFB3
ENG	0.820	2.58E-104	MFB3
TINAGL1	0.691	8.12E-103	MFB3
OGN	1.372	3.03E-101	MFB3
CRIM1	0.922	1.45E-97	MFB3
LTC4S	0.728	5.03E-86	MFB3
PTGS2	1.134	3.03E-79	MFB3
CLU	0.902	1.86E-78	MFB3
TCF4	0.675	2.81E-75	MFB3
EFEMP1	0.623	3.63E-70	MFB3
SPARCL1	0.582	1.04E-66	MFB3
ART4	0.572	1.27E-66	MFB3
COL8A1	0.796	1.15E-58	MFB3
PCDH7	0.557	4.15E-57	MFB3
OMD	0.688	4.56E-57	MFB3
MYH10	0.617	7.13E-56	MFB3
CRTAC1	0.762	9.81E-53	MFB3
ADH1B	0.692	2.47E-51	MFB3

© 2022. Shi X et al. Published online at http://www.aginganddisease.org/EN/10.14336/AD.2022.0313

EDN1	1.108	6.99E-46	MFB3
CP	0.676	4.09E-45	MFB3
SAA1	1.615	2.13E-44	MFB3
ASPN	0.604	1.29E-38	MFB3
CXCL2	1.089	2.16E-37	MFB3
EGFL7	0.540	5.71E-32	MFB3
ANKRD1	0.509	2.82E-29	MFB3
THBD	0.583	2.07E-27	MFB3
IER3	0.530	3.16E-25	MFB3

© 2022. Shi X et al. Published online at http://www.aginganddisease.org/EN/10.14336/AD.2022.0313
